# The Phosphatase Cascade Nem1/Spo7-Pah1 Regulates Fungal Development, Lipid Homeostasis, and Virulence in Botryosphaeria dothidea

**DOI:** 10.1128/spectrum.03881-22

**Published:** 2023-05-16

**Authors:** Weichao Ren, Yihan Zhang, Meiqi Zhu, Zequn Liu, Sen Lian, Caixia Wang, Baohua Li, Na Liu

**Affiliations:** a Key Laboratory of Integrated Crop Pest Management of Shandong Province, College of Plant Health and Medicine, Qingdao Agricultural University, Qingdao, China; University of Natural Resources and Life Sciences Vienna

**Keywords:** *Botryosphaeria dothidea*, phosphatase, development, lipid homeostasis, virulence

## Abstract

Protein phosphatase complex Nem1/Spo7 plays crucial roles in the regulation of various biological processes in eukaryotes. However, its biological functions in phytopathogenic fungi are not well understood. In this study, genome-wide transcriptional profiling analysis revealed that Nem1 was significantly upregulated during the infection process of Botryosphaeria dothidea, and we identified and characterized the phosphatase complex Nem1/Spo7 and its substrate Pah1 (a phosphatidic acid phosphatase) in B. dothidea. Nem1/Spo7 physically interacted with and dephosphorylated Pah1 to promote triacylglycerol (TAG) and subsequent lipid droplet (LD) synthesis. Moreover, the Nem1/Spo7-dependently dephosphorylated Pah1 functioned as a transcriptional repressor of the key nuclear membrane biosynthesis genes to regulate nuclear membrane morphology. In addition, phenotypic analyses showed that the phosphatase cascade Nem1/Spo7-Pah1 was involved in regulating mycelial growth, asexual development, stress responses, and virulence of *B. dothidea*.

**IMPORTANCE** Botryosphaeria canker and fruit rot caused by the fungus *Botryosphaeria dothidea* is one of the most destructive diseases of apple worldwide. Our data indicated that the phosphatase cascade Nem1/Spo7-Pah1 plays important roles in the regulation of fungal growth, development, lipid homeostasis, environmental stress responses, and virulence in *B. dothidea*. The findings will contribute to the in-depth and comprehensive understanding of Nem1/Spo7-Pah1 in fungi and the development of target-based fungicides for disease management.

## INTRODUCTION

The ascomycete fungus Botryosphaeria dothidea is a major pathogen causing Botryosphaeria canker and fruit rot, which is among the most devastating diseases affecting apple crops globally (1–3). B. dothidea infects branches and fruits in both pre- and postharvest phases, resulting in considerable reduction of apple yields and quality (4). Currently, fungicide applications are the main control measure for this disease due to the lack of *B. dothidea*-resistant apple cultivars. However, the effectiveness of a great many widely used fungicides has been threatened by the emergence of resistant pathogen populations in the field after long-term intensive application (5–7). Therefore, there is an urgent need to seek alternative strategies for controlling apple ring rot. Fully understanding the fundamental biology of *B. dothidea* will facilitate breeding for disease resistance and development of novel fungicides, establishing a more efficient disease management strategy.

Reversible phosphorylation governed by protein kinases and phosphatases plays vital roles in regulating diverse biological processes in eukaryotes (8). Based on the amino acid sequences at active sites, protein phosphatases could be generally grouped into three major categories, including serine/threonine (S/T) protein phosphatases with active-site sequence DXH(X)nDXXD(X)nNH(D/E), tyrosine phosphatases with active site sequence C(X)_5_R, and a later-emerging haloacid dehalogenase (HAD) family with the DXDX(T/V) active-site sequence (9–11).

Nem1 (nuclear envelope morphology protein 1), a HAD family phosphatase with catalytic motif DXDX(T/V), is evolutionally conserved throughout eukaryotes. In yeasts, the function of Nem1 in lipid metabolism has been widely studied (12–14). As the catalytic subunit, Nem1 forms a phosphatase holoenzyme with its regulatory subunit Spo7 (sporulation-specific protein 7), and the Nem1/Spo7 complex is involved in regulating nuclear/endoplasmic reticulum (ER) membrane association of Pah1, a phosphatidic acid (PA) phosphatase (15–19). Pah1 is phosphorylated by multiple kinases and dephosphorylated by Nem1/Spo7 complex (20–24); the dephosphorylated Pah1 is active and then dephosphorylates PA to produce diacylglycerol (DAG), which is the precursor of phospholipids through the *de novo* (Kennedy) pathway and also is required for the formation of triacylglycerols (TAGs), the most prominent neutral lipids in lipid droplets (LDs) (12, 25). In addition, disruption of yeast Nem1/Spo7 complex results in defects in sporulation, premeiotic replication, and autophagy (19, 26, 27). In Cryptococcus neoformans, which causes life-threatening meningoencephalitis mainly in immunocompromised patients, the genome-wide functional analysis of phosphatases revealed that Nem1 plays important roles in melanin production, multiple stresses responses, and virulence (28). Tetrahymena thermophila contains four Nem1 homologues, which are in involved in regulating LD biogenesis via Pah1 (29). NEP1-R1 (Spo7 homologue) knockdown in Caenorhabditis elegans embryos results in twinned nuclei, similar to depletion of C. elegans CTDNEP1 (Nem1 homologue) (30). Dullard (Nem1 homologue) is essential for neural development of *Xenopus* (31).

However, to date, only a few systematic studies regarding the biological functions of Nem1/Spo7 complex have been reported for filamentous fungi. Functional analysis of the phosphatome in Aspergillus fumigatus preliminarily showed that NemA is involved in regulating responses to stresses mediated by a cell wall-damaging agent and iron starvation (32). In phytopathogenic fungi, Nem1/Spo7 complex regulates LD biogenesis, disruption of the complex caused pleiotropic defects on hyphal growth, mycotoxin production, oxidation stress response, and pathogenicity in Fusarium graminearum (33, 34). In Magnaporthe oryzae, only Nem1 and not Spo7 has been identified, and Nem1 is important for mycelial growth and conidiation but has no effect on host infection (35).

In this study, genome-wide transcriptional profiles analysis showed that the Nem1 homologue in *B. dothidea* was significantly upregulated and predicted to play an important role during the infection process. Therefore, a deep understanding of the fundamental biology of Nem1/Spo7 complex in *B. dothidea* will promote the development of new fungicides for apple ring rot management. We identified and dissected the biological functions of the HAD family phosphatase complex Nem1/Spo7 and its substrate Pah1 in *B. dothidea*. Our studies demonstrated that the PA phosphatase Pah1 interacted with and was dephosphorylated by Nem1/Spo7 and functioned as a transcriptional repressor of key nuclear membrane biosynthesis genes to regulate nuclear membrane morphology in *B. dothidea*. Moreover, dephosphorylated Pah1 promoted TAG and subsequent LD biosynthesis. In addition, Δ*Nem1*, Δ*Spo7*, and Δ*Pah1* mutants displayed obvious defects in vegetative growth, asexual development, stress responses, and virulence *in planta*. Collectively, the results show that phosphatase cascade Nem1/Spo7-Pah1 was important for hyphal growth, cellular development, lipid metabolism, and pathogenicity in *B. dothidea*.

## RESULTS

### Identification and sequence analysis of Nem1 and Spo7 in *B. dothidea*.

**FIG 1:**
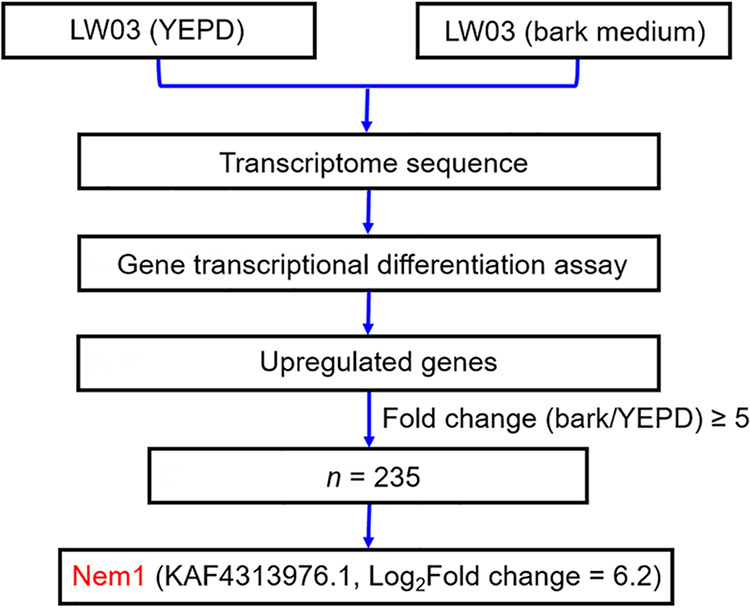
Nem1 was upregulated during the infection process of *B. dothidea*, analyzed by comparative transcriptome assays.

**FIG 2:**
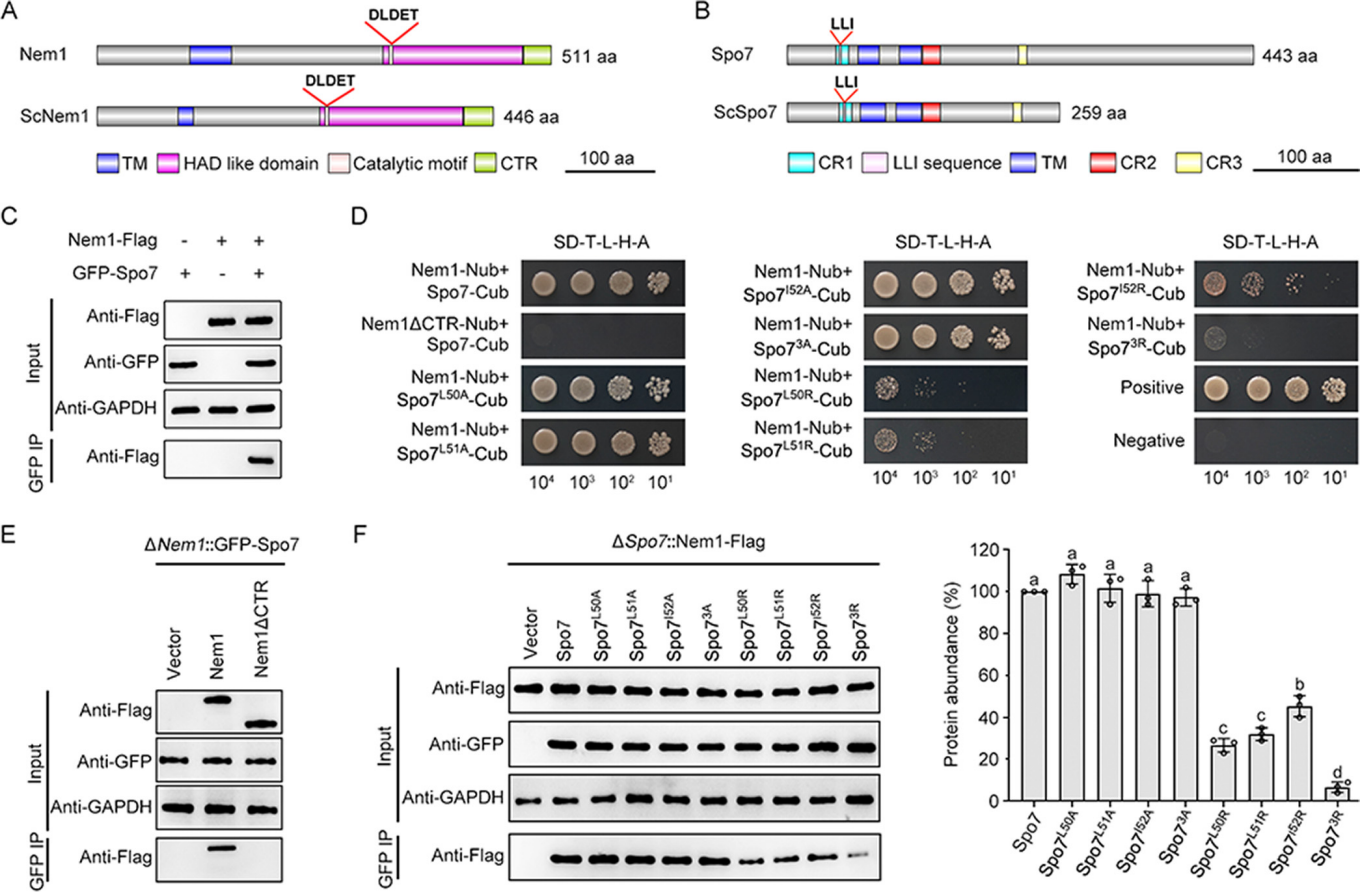
Nem1 interacts with Spo7. Shown is the schematic architecture of the Nem1 (A) and Spo7 (B) proteins in *B. dothidea*. S. cerevisiae Nem1 (ScNem1) and Spo7 (ScSpo7) were selected as references. Conserved domains are indicated. TM, transmembrane domain; CTR, C-terminal region; CR, conserved region. (C) Coimmunoprecipitation (co-IP) assays of interaction between Nem1 and Spo7. (D) Split-ubiquitin membrane-based yeast two-hybrid analysis of interaction patterns among various protein pairs. (E) Co-IP analysis of interaction between Spo7 and Nem1 lacking the CTR. (F) Co-IP assays of interaction among Nem1 and Spo7 containing various amino acid mutations (left). The relative intensities of the Nem1-Flag band in GFP IP of each strain were quantified (right). The intensity of the Nem1-Flag band in the strain containing wild-type Spo7 was set as 100%. Error bars represent standard errors of three independent experiments, and values on the bars followed by the same letter are not significantly different according to a Fisher’s least significant difference (LSD) test at a *P* value of 0.05.

To identify candidate fungicide target proteins important for pathogenicity of *B. dothidea*, we performed genome-wide transcriptome analysis of the wild-type (WT) strain LW03 cultured in yeast extract-peptone-dextrose (YEPD) for 24 h with or without transference into liquid bark medium (to simulate infection conditions) for 12 h. Gene expression profile analysis revealed that Nem1 of *B. dothidea* encoded by KAF4313976.1 (NCBI:protein accession number KAF4313976.1) was upregulated about 74-fold in hyphae with transference into liquid bark medium compared to the hyphae in YEPD alone (Fig. 1), suggesting that the phosphatase Nem1 in *B. dothidea* may play crucial roles during infection process. Next, we retrieved the Spo7 orthologue of *B. dothidea* by BLAST search of the *B. dothidea* genome database (https://www.ncbi.nlm.nih.gov/biosample/SAMN13735646) with the Saccharomyces cerevisiae Spo7 protein sequence as a query. Sequence analysis revealed that *NEM1* and *SPO7* of *B. dothidea* are predicted to encode proteins with 511 and 443 amino acids (aa), sharing 48% and 30% sequence similarity with yeast Nem1 and Spo7, respectively. Phylogenetic analysis by IQTREE (http://www.iqtree.org/) of Nem1/Spo7 proteins in *B. dothidea* and other organisms indicated that the phosphatase catalytic subunit Nem1 and its regulatory subunit Spo7 are evolutionarily conserved from yeast to human (see Fig. S1A and B in the supplemental material). Structural analysis showed that Nem1 contains a transmembrane (TM) domain, a C-terminal region (designated CTR), and a typical catalytic HAD domain with the DLDET catalytic motif, which is highly conserved in S. cerevisiae (Fig. 2A). Spo7 possesses three conserved regions (CR1 to CR3), two TM domains, and a conserved hydrophobic Leu-Leu-Ile (LLI) sequence comprising residues 50 to 52 in CR1 (Fig. 2B). In addition, yeast complementation assays showed that *B. dothidea NEM1* could not restore the growth defect of yeast *NEM1* deletion mutant on yeast extract peptone raffinose galactose (YPRG) medium containing caffeine (Fig. S2), indicating that the function of Nem1 at least partially differs between yeast and *B. dothidea*.

### Nem1 interacts with Spo7 to form phosphatase complex Nem1/Spo7.

To determine the interaction relations between Nem1 and Spo7 of *B. dothidea*, we conducted a coimmunoprecipitation (co-IP) analysis. Briefly, the total proteins were isolated from the wild-type strain (LW03) transformed with Nem1-3×Flag and/or green fluorescent protein (GFP)-Spo7, and proteins copurifying with the anti-GFP agarose were analyzed by Western blotting with the monoclonal anti-Flag antibody. As shown in Fig. 2C, Nem1 interacted with Spo7 in the co-IP assay. In addition, split-ubiquitin membrane-based yeast two-hybrid assays were performed to further confirm the association between Nem1 and Spo7. As shown in Fig. 2D, Nem1 could directly interact with Spo7. Moreover, the absence of CTR in Nem1 (Nem1ΔCTR) completely abolished the interaction between Nem1 and Spo7 (Fig. 2D and E). To determine the effects of the hydrophobicity of the LLI sequence in Spo7 on the interaction pattern among Nem1 and Spo7, we individually or simultaneously mutated the three amino acids to alanine (A) and arginine (R); the resulting proteins were called Spo7^L50A^, Spo7^L51A^, Spo7^I52A^, Spo7^3A^, Spo7^L50R^, Spo7^L51R^, Spo7^I52R^, and Spo7^3R^, respectively. Alanine conserves the hydrophobic property, while arginine conserves the hydrophilic property. Co-IP analysis showed that the alanine-substituted forms of Spo7 (i.e., Spo7^L50A^, Spo7^L51A^, Spo7^I52A^, and Spo7^3A^) exhibited a level of interaction with Nem1 similar to that of wild-type Spo7, while the interaction levels of the arginine-substituted forms Spo7^L50R^, Spo7^L51R^, Spo7^I52R^, and Spo7^3R^ were significantly reduced (72, 68, 55, and 93%, respectively) (Fig. 2F). The split-ubiquitin membrane-based yeast two-hybrid assays showed consistent results (Fig. 2D). These results indicate that Nem1 interacts with Spo7 to form a phosphatase complex in *B. dothidea*. The CTR of Nem1 and the hydrophobicity of the LLI sequence in Spo7 are crucial for interaction between Nem1 and Spo7.

### Identification of the phosphatidic acid phosphatase Pah1.

**FIG 3:**
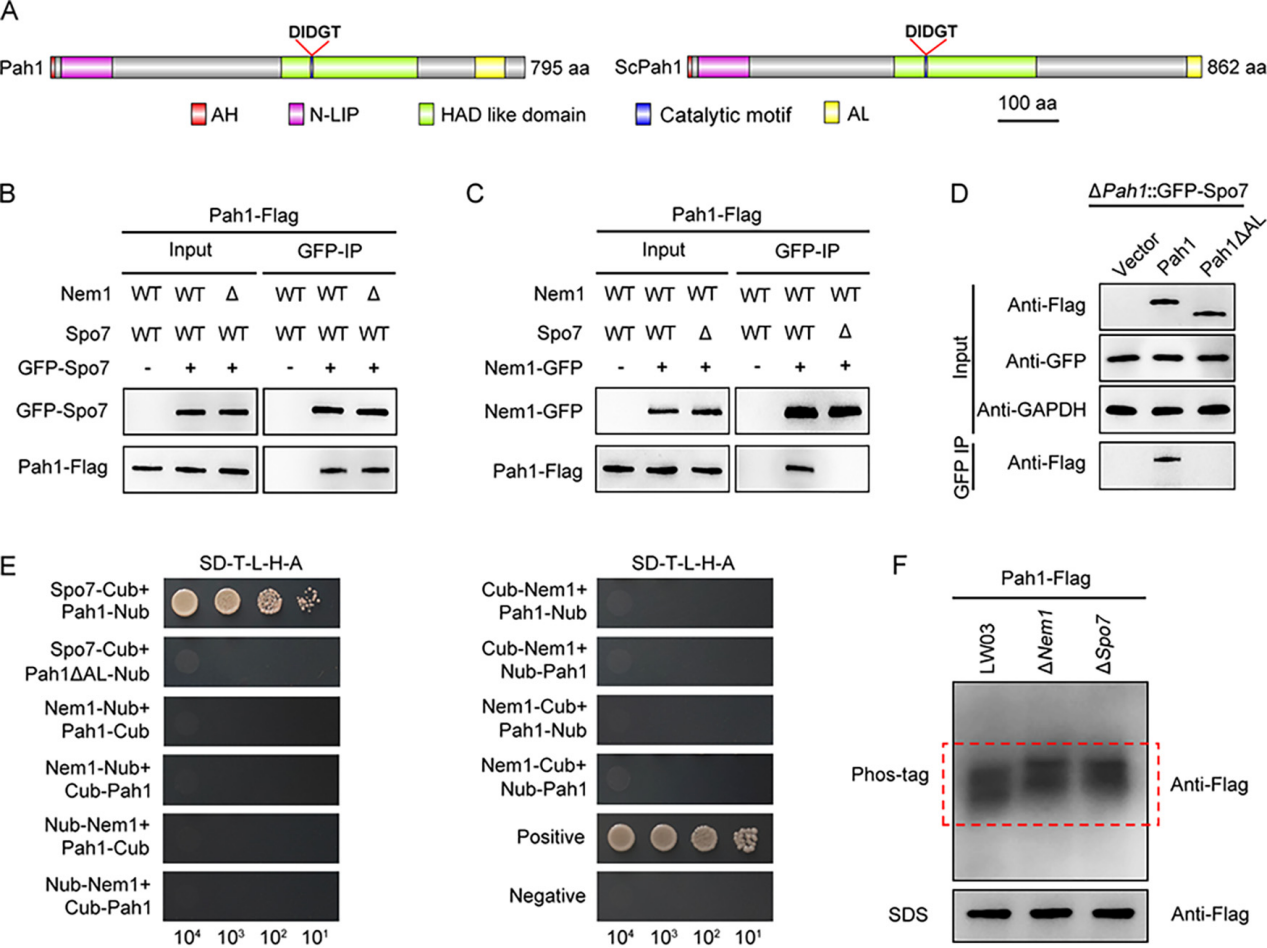
Phosphatase complex Nem1/Spo7 interacts with and dephosphorylates Pah1. (A) Schematic architecture of the Pah1 proteins in *B. dothidea* and S. cerevisiae (ScPah1). Conserved domains are indicated. AH, amphipathic helix domain; AL, acidic tail sequence. (B) Co-IP assays revealed that loss of Nem1 had no effect on the interaction between Spo7 and Pah1. (C) Co-IP assays showed that Spo7 was essential for interaction between Nem1 and Pah1. (D) Pah1 physically interacted with Spo7 by its AL domain in co-IP assays. (E) Split-ubiquitin membrane-based yeast two-hybrid analysis of the interaction between Nem1, Spo7, and wild type or mutated forms of Pah1. (F) Nem1/Spo7 complex was essential for dephosphorylation of Pah1. The Pah1-Flag protein from the wild-type strain (LW03) or the Δ*Nem1* or Δ*Spo7* mutant was subjected to Phos-tag sodium dodecyl sulfate-polyacrylamide gel electrophoresis (SDS-PAGE; top) and normal SDS-PAGE (bottom), followed by immunoblotting with anti-Flag antibody.

To identify the substrate of Nem1/Spo7 complex, we sought candidate proteins which interact with Nem1/Spo7 by conducting affinity capture mass spectrometry (MS) as described in Materials and Methods. In this assay, we found that protein encoded by KAF4301686.1 (NCBI:protein accession number KAF4301686.1) was copurified with both Nem1-GFP and GFP-Spo7 (Table S1). Bioinformatics and phylogenic analysis showed that the KAF4301686.1-encoded protein is homologous to S. cerevisiae phosphatide phosphatase Pah1 (Fig. S1C), and it is therefore designated Pah1 of *B. dothidea*. *PAH1* is predicted to encode a protein with 795 aa, sharing 54% sequence similarity with yeast Pah1. Structural analysis showed that Pah1 consists of a conserved N-LIP domain and HAD-like domain with the DIDGT catalytic motif, which are required for PA phosphatase activity. In addition, Pah1 contained a conserved amphipathic helix (AH) domain and an acidic tail (AL, aspartate/glutamate-rich sequences) in the N terminus and C terminus, respectively (Fig. 3A).

### Nem1/Spo7 complex interacts with and dephosphorylates Pah1.

To verify the interactions between Pah1 and Nem1/Spo7 complex in *B. dothidea*, co-IP assays were conducted, and the results showed that both Nem1 and Spo7 interacted with Pah1; Nem1 was not necessary for the interaction among Pah1 and Spo7 (Fig. 3B), while loss of Spo7 resulted in the complete abolishment of interaction between Pah1 and Nem1 (Fig. 3C). Furthermore, AL of Pah1 was essential for its interaction with Spo7 (Fig. 3D). The interaction patterns between Nem1/Spo7 complex and Pah1 were further examined by the split-ubiquitin membrane-based yeast two-hybrid assays. Consistent with the results of the co-IP assays, Nem1 only physically interacted with Spo7 and not with Pah1, while Spo7 directly interacted with both Nem1 and Pah1, and lack of AL in Pah1 abolished its interaction with Spo7 (Fig. 3E). Collectively, these results implied that Nem1/Spo7 complex physically interacted with Pah1.

Next, to confirm whether Pah1 is the catalytic substrate of Nem1/Spo7 complex in *B. dothidea*, we first generated deletion mutants of *NEM1* (Δ*Nem1*) and *SPO7* (Δ*Spo7*) using a homology recombination strategy (Fig. S3A and B) and then determined the phosphorylation patterns of Pah1 in wild-type strain LW03 and Δ*Nem1* and Δ*Spo7* mutants by Phos-tag assays as described in Materials and Methods. Briefly, the Pah1-Flag fusion construct was transformed into LW03 and the Δ*Nem1* and Δ*Spo7* mutants and the mobility shifts of Pah1-Flag in the resulting strains (LW03::Pah1-Flag, Δ*Nem1*::Pah1-Flag, and Δ*Spo7*::Pah1-Flag) were tested by immunoblotting assay using an anti-Flag antibody. As shown in Fig. 3F, the intensity of fast-migrating bands, which are correlated with dephosphorylated isoforms of Pah1, were significantly decreased after deletion of *NEM1* or *SPO7*, indicating that Pah1 was dephosphorylated by Nem1/Spo7 complex in *B. dothidea*.

### The Nem1/Spo7-Pah1 cascade regulates vegetative growth and asexual development.

To test the detailed functions of the Nem1/Spo7-Pah1 cascade in *B. dothidea*, we generated single-deletion or complementation mutants of the cascade (Δ*Nem1*, Δ*Spo7*, Δ*Pah1*, Δ*Nem1-C*, Δ*Spo7-C*, and Δ*Pah1-C*) as described in Materials and Methods, and the mutants were confirmed by PCR and Western blotting assays (Fig. S3).

**FIG 4:**
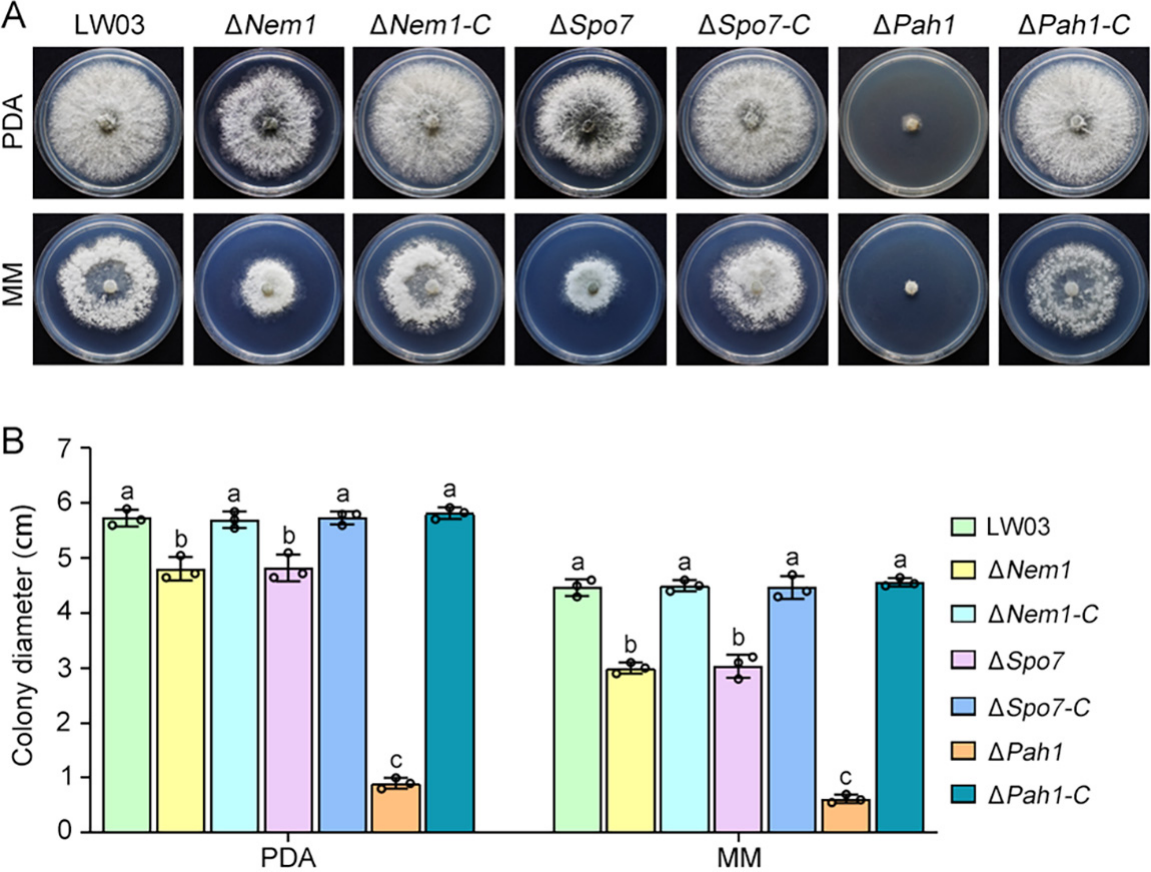
Single deletion of Nem1, Spo7, or Pah1 impairs hyphal growth. (A) Colony morphology of each strain. LW03 (wild-type strain), Δ*Nem1*, Δ*Nem1*-*C*, Δ*Spo7*, Δ*Spo7-C*, and Δ*Pah1* mutants, and the Δ*Pah1-C* complemented strain were grown on potato dextrose agar (PDA) and MM at 25°C for 3 days. (B) Average colony diameter of each strain after incubation at 25°C on PDA and MM for 3 days. Error bars represent standard errors of three independent experiments, and values on the bars followed by the same letter are not significantly different according to LSD test at a *P* value of 0.05.

To determine the role of Nem1/Spo7-Pah1 in mycelial growth, LW03, Δ*Nem1*, Δ*Spo7*, and Δ*Pah1* mutant, and complemented strains were cultured on potato dextrose agar (PDA) and minimal medium (MM). As shown in Fig. 4, after incubation at 25°C for 3 days, the Δ*Nem1* mutant exhibited a colony morphology similar to that of the Δ*Spo7* mutant, which grow slower on PDA and MM than LW03 and their complemented strains (with growth reductions of 17% and 27% on PDA and MM, respectively). Compared with the Δ*Nem1* and Δ*Spo7* mutants, the Δ*Pah1* mutant displayed a significantly more serious defect in hyphal growth rate, which was decreased to 14% of that of LW03.

**FIG 5:**
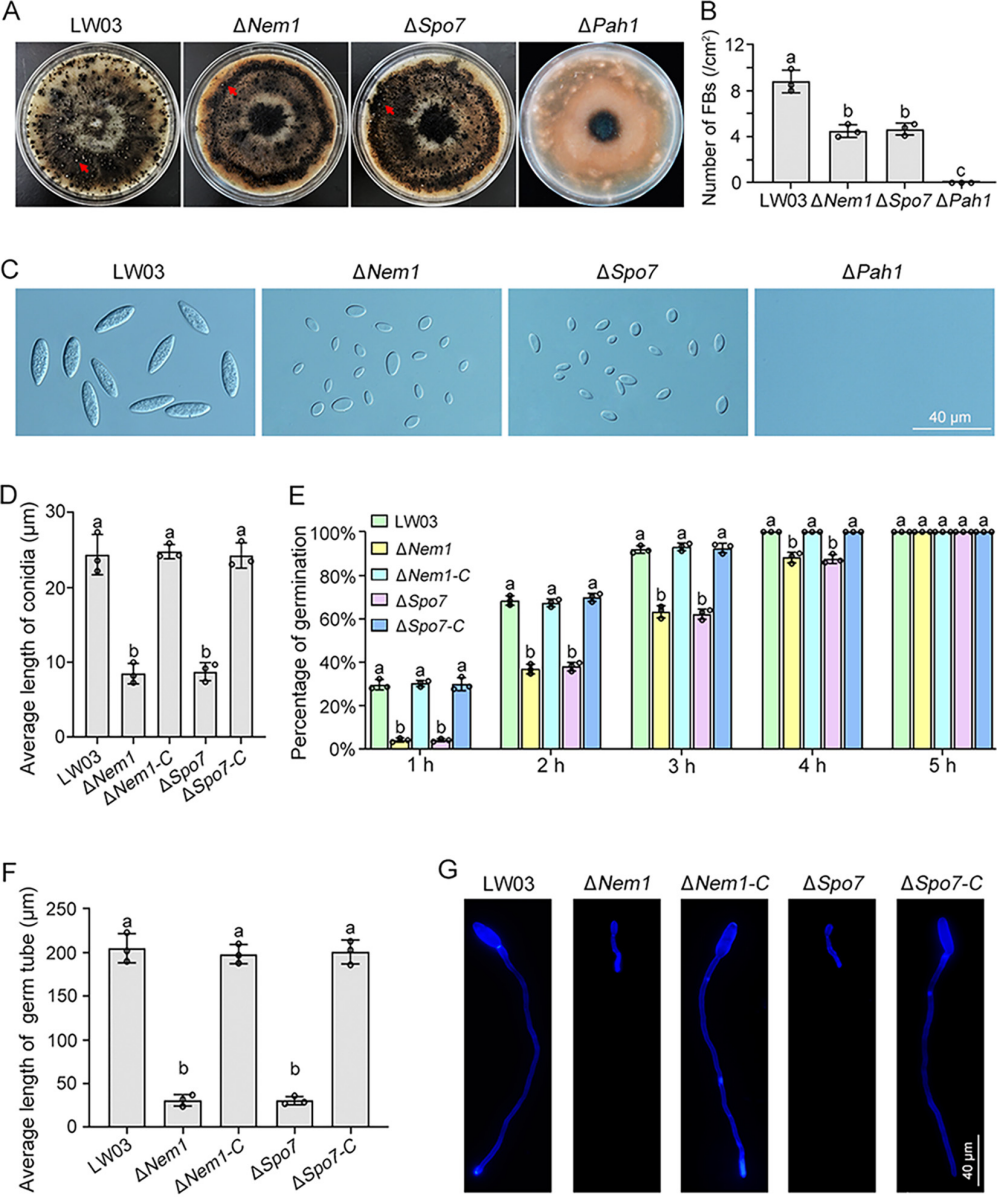
The Nem1/Spo7-Pah1 cascade regulates asexual development. (A) Fruiting bodies (FBs) formed on carrot agar medium of each strain. FBs (indicated by red arrows) generated by the LW03, Δ*Nem1*, Δ*Spo7*, and Δ*Pah1* strains were assayed after incubation at 25°C for 2 weeks. (B) Average numbers of FBs produced by the LW03, Δ*Nem1*, Δ*Spo7*, and Δ*Pah1* strains. (C) Conidial morphology of each strain. (D) The average length of conidia generated by each strain was quantified. (E) Percentage of germinated conidia from each strain. Conidia of each strain were suspended in 2% sucrose solution, and conidial germination of 100 conidia was examined at the indicated time points. (F) The average length of 100 germ tubes of conidia from each strain was determined after incubation in 2% sucrose solution for 4 h. Error bars represent standard errors of three replicated experiments, and values on the bars followed by the same letter are not significantly different according to LSD test at a *P* value of 0.05. (G) After incubation in 2% glucose for 4 h, germinated conidia of each strain were stained with calcofluor white (CFW) and examined under a microscope.

Asexual reproduction plays important roles in the life cycle of *B. dothidea* and epidemic apple ring rot. To investigate the roles of the Nem1/Spo7-Pah1 cascade in asexual development, conidial production by each strain was evaluated on carrot agar conidium induction medium. After 7 days of incubation with black light exposure at 25°C, no fruiting bodies (FBs) were generated by the Δ*Pah1* mutant, and the Δ*Nem1* mutant produced numbers of FBs similar to those produced by the Δ*Spo7* mutant, which were obviously fewer than that of LW03 and complemented strains (Fig. 5A and B and Fig. S4). Moreover, the conidia in FBs generated by the Δ*Nem1* and Δ*Spo7* mutants exhibited abnormal morphologic characteristics (Fig. 5C). The average length of conidia produced by the Δ*Nem1* and Δ*Spo7* mutants were much shorter than those of LW03 and complemented strains (Fig. 5D). In addition, the abnormal conidia of the Δ*Nem1* and Δ*Spo7* mutants exhibited a lower germination rate and shorter germinal tubes than those of the LW03 and complemented strains in the presence of 2% (wt/vol) sucrose (Fig. 5E to G). Collectively, the results showed that the Nem1/Spo7-Pah1 cascade of *B. dothidea* is important for hyphal growth and asexual development.

### The Nem1/Spo7-Pah1 cascade plays crucial roles in LD accumulation.

**FIG 6:**
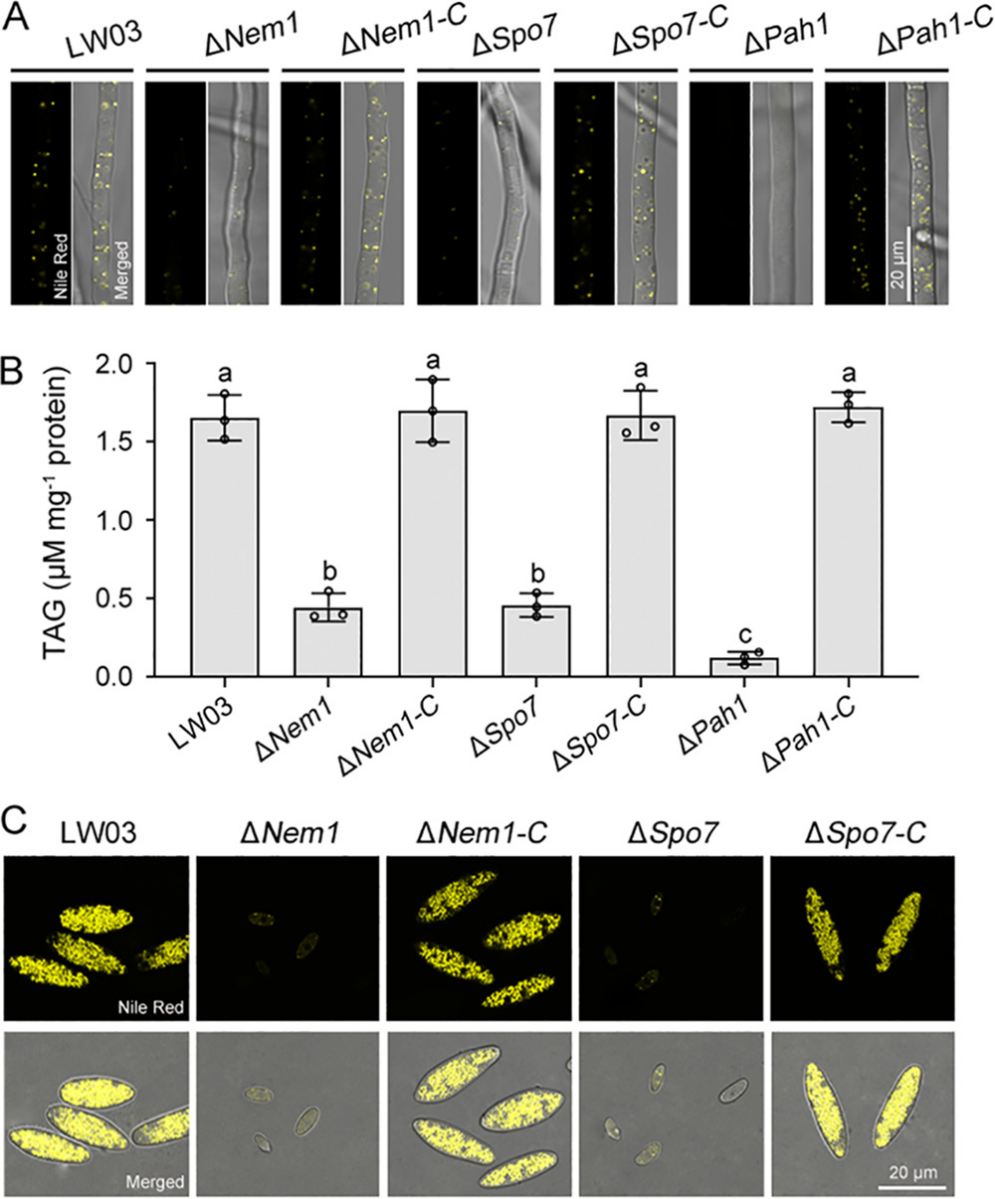
The Nem1/Spo7-Pah1 cascade plays important roles in lipid droplet (LD) and triacylglycerol (TAG) biosynthesis. (A) TLD accumulation patterns in hyphae of each strain. The yellow bodies (representing LDs) in hyphae were stained with Nile red. (B) Quantification of TAG contents in the mycelia of each strain. Error bars represent standard errors of three replicated experiments, and values on the bars followed by the same letter are not significantly different according to LSD test at a *P* value of 0.05. (C) LDs in conidia of each strain were stained with Nile red.

To elucidate the functions of the Nem1/Spo7-Pah1 cascade in lipid droplet (LD) biogenesis, we observed the accumulation patterns of LDs using the LD staining dyes Nile red and BODIPY (boron-dipyrromethene). As shown in Fig. 6A and Fig. S5A, histochemical analysis showed that no visible LDs were observable in the hyphae of the Δ*Pah1* mutant, and the Δ*Nem1* and Δ*Spo7* mutants contained much fewer LDs than LW03 and complemented strains. In yeast, dephosphorylated Pah1 upon Nem1/Spo7 complex catalyzes PA to produce DAG, which is then acylated to produce TAGs, the most prominent neutral lipids in LDs (36). Therefore, TAG quantification was performed and results showed that the TAG contents in the mycelia of the three deletion mutants (Δ*Nem1*, Δ*Spo7*, and Δ*Pah1*) were reduced to very low values, of which the Δ*Pah1* mutant decreased the most (Fig. 6B). In addition, we tested the LD accumulation patterns in conidia of each strain. As shown in Fig. 6C and Fig. S5B, the LD number was seriously decreased in conidia of the Δ*Nem1* and Δ*Spo7* mutants compared with those in LW03 and complemented strains. Overall, these results indicate that the Nem1/Spo7-Pah1 cascade plays crucial roles in TAG and LD synthesis.

### Dephosphorylated Pah1 upon Nem1/Spo7 functions as a transcriptional repressor to regulate nuclear envelope morphology.

**FIG 7:**
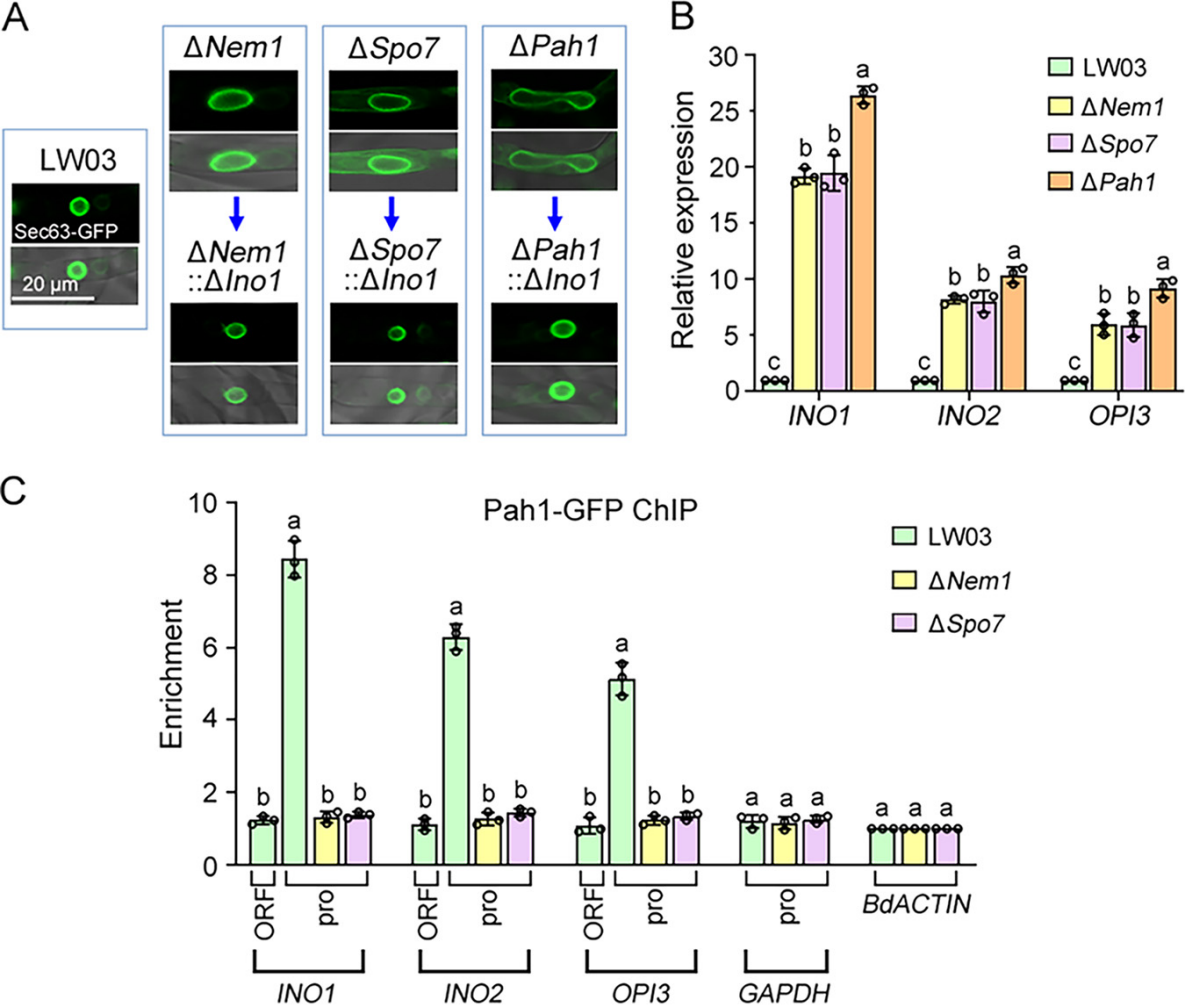
Dephosphorylated form of Pah1 upon Nem1/Spo7 functions as a transcriptional repressor to regulate nuclear membrane organization. (A) Morphology of nuclear membrane in each strain. Sec63-GFP was selected to visualize nuclear membrane structure in LW03, Δ*Nem1*, Δ*Spo7*, and Δ*Pah1* strains and Δ*Nem1*::Δ*Ino1*, Δ*Spo7*::Δ*Ino1*, and Δ*Pah1*::Δ*Ino1* double-deletion mutants. (B) Transcript levels of three key phospholipid biosynthesis genes (*INO1*, *INO2*, and *OPI3*) in LW03, Δ*Nem1*, Δ*Spo7*, and Δ*Pah1* strains after 36 h of incubation of each strain in liquid YEPD medium. Error bars represent standard errors of three replicated experiments, and values on the bars followed by the same letter are not significantly different according to LSD test at a *P* value of 0.05. (C) Chromatin immunoprecipitation-quantitative PCR (ChIP-qPCR) assays revealed that Pah1 associated with the promoters of *INO1*, *INO2*, and *OPI3*, and Nem1/Spo7 complex was essential for the association. The *ACTIN* was used as background control and given the value 1.

During the process of protoplast preparation for mutant complementation, we observed that the size of protoplasts produced by the Δ*Nem1*, Δ*Spo7*, and Δ*Pah1* mutants were obviously bigger than those of LW03 and complemented strains (Fig. S6), suggesting the phosphatase cascade Nem1/Spo7-Pah1 may be involved in regulating protoplast membrane growth. Previous studies revealed that yeast Pah1 is a key regulator of nuclear membrane growth during the cell cycle. To investigate the functions of the *B. dothidea* Nem1/Spo7-Pah1 cascade in regulating nuclear membrane morphology, the nuclear membrane reporter *B. dothidea* Sec63 fused with GFP was transferred into LW03 and the Δ*Nem1*, Δ*Spo7*, and Δ*Pah1* mutants. As shown in Fig. 7A, the Δ*Pah1* mutant exhibited seriously enlarged and irregularly shaped nuclei, and the Δ*Nem1* and Δ*Spo7* mutants showed a consistent but milder defect on nuclear envelope morphology compared with the Δ*Pah1* mutant. The major lipid components of the nuclear membrane in yeast are phosphatidylinositol (PI) and phosphatidylcholine (PC). Ino1 is the rate-limiting enzyme for PI synthesis, and Opi3 is the enzyme catalyzing the final steps in the production of PC (18). To elucidate the mechanism of Nem1/Spo7-Pah1 in regulating nuclear membrane expansion in *B. dothidea*, quantitative real-time PCR (qRT-PCR) experiments were performed. As shown in Fig. 7B, the mRNA levels of *INO1*, its transcriptional activator *INO2*, and *OPI3* showed a significant upregulation in the Δ*Nem1*, Δ*Spo7*, and Δ*Pah1* mutants with respect to LW03. Moreover, the increase in the mRNA levels was higher in the Δ*Pah1* mutant than in the Δ*Nem1* or Δ*Spo7* mutant, consistent with the phenomenon that the Δ*Pah1* mutant displayed more severe nuclear membrane expansion than the Δ*Nem1* or Δ*Spo7* mutant. In addition, deletion of *INO1* (whose mRNA levels increased most) obviously repressed nuclear membrane expansion in most cells of the Δ*Nem1*, Δ*Spo7*, and Δ*Pah1* mutants (Fig. 7A), implying that dephosphorylation of Pah1 upon Nem1/Spo7 complex affects nuclear envelope morphology by controlling the expression of key nuclear membrane synthesis genes.

To further explore whether dephosphorylated Pah1 could bind to the promoters and function as a transcriptional regulator of nuclear membrane synthesis genes, we performed chromatin immunoprecipitation (ChIP) of the Pah1-GFP fusion, followed by qRT-PCR analysis. The results showed that Pah1 in fact associated with promoters but not the open reading frames (ORFs) of *INO1*, *INO2*, and *OPI3*, and the enriched amounts of Pah1 in these promoters were approximately 4.5- to 7.5-fold over the background levels detected at the promoter of a reference gene, *GAPDH* (encoding glyceraldehyde-3-phosphate dehydrogenase). What is more, the association of Pah1-GFP with promoters of the three genes was disrupted in the Δ*Nem1* and Δ*Spo7* strains (Fig. 7C). These results suggest that dephosphorylated Pah1 by Nem1/Spo7 complex functions as a transcriptional repressor of nuclear membrane biosynthesis genes to maintain normal nuclear morphology in *B. dothidea*.

### The Nem1/Spo7-Pah1 cascade is important for full virulence of *B. dothidea*.

**FIG 8:**
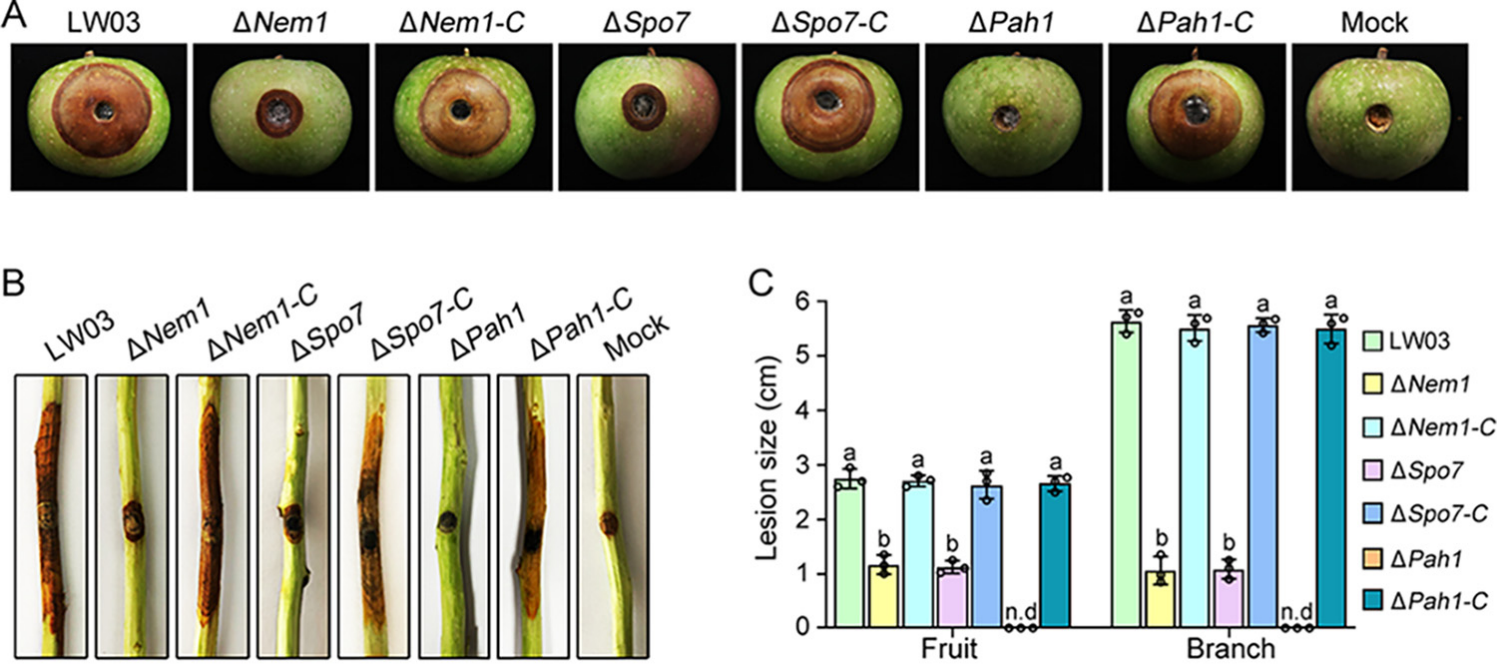
The Nem1/Spo7-Pah1 cascade is required for full virulence. (A) Disease symptoms on young apple fruits following inoculation with the wild-type strain LW03, the Δ*Nem1*, Δ*Spo7*, and Δ*Pah1* mutants, and complementation strains for 7 days. (B) Disease symptoms on 1-year-old apple branches 10 days after inoculation of each strain. (C) Lesion size on apple fruits and branches caused by each strain.

*B. dothidea* infects both branches and fruits of apples. To determine the role of the phosphatase cascade Nem1/Spo7-Pah1 in the virulence of *B. dothidea*, we evaluated the pathogenic ability of the Δ*Nem1*, Δ*Spo7*, and Δ*Pah1* mutants on young Fuji apple fruits and 1-year-old Fuji twigs. As shown in Fig. 8, 7 days after inoculation, the wild-type strain LW03 and complemented strains caused serious typical ring rot symptoms on apple fruits. However, under the same conditions, the lesion size of both the Δ*Nem1* and Δ*Spo7* mutants were significantly smaller. Consistently, the Δ*Nem1* and Δ*Spo7* mutants were also obviously defective in infection of excised apple twigs 10 days after inoculation. The Δ*Pah1* mutant was unable to cause any typical symptoms on both apple fruits and twigs. To test whether the attenuated virulence of the Δ*Nem1* and Δ*Spo7* mutants was caused by their growth defect, the colony morphology of each strain was tested on apple fruit and bark media, which were similar to the nutritional conditions during the infection process. As shown in Fig. S7, the amounts of aerial mycelia of the Δ*Nem1* and Δ*Spo7* mutants were similar to those of LW03 and complemented strains, and the growth rates of deletion mutants were decreased slightly on both fruit and bark media. These results suggest that the main reason for the reduced pathogenicity of the Δ*Nem1* and Δ*Spo7* mutants is not growth defect. Previous studies showed that plants produced phytoalexin such as benzoxazolin-2-one (BOA) during the defense response to various biotic stresses (37). In this study, we found that both the Δ*Nem1* and Δ*Spo7* mutants displayed increased sensitivity toward *O*-aminophenol (BOA detoxification intermediate) compared with LW03 and complemented strains (Fig. S8). These results suggest that the phosphatase cascade Nem1*/*Spo7-Pah1 was important for pathogenicity of *B. dothidea*, and Nem1*/*Spo7 complex regulates virulence at least partially by overcoming phytoalexin produced by plants during the infection process.

## DISCUSSION

Nem1, a HAD family phosphatase number, serves as the catalytic subunit, which binds to its regulatory subunit Spo7 to form phosphatase complex Nem1/Spo7, has been reported to play critical roles in various biological processes in eukaryotes (18, 27, 38, 39). However, the function of Nem1/Spo7 (CTDNEP1-NEP1R1 in mammalian cells) complex in filamentous fungi remains obscure. In this study, we found that the Nem1 homologue in *B. dothidea* was significantly upregulated during the infection process, implying that Nem1 is important for pathogenicity of *B. dothidea*, which prompted us to further investigate the biological functions of Nem1/Spo7 complex in *B. dothidea*.

We identified the Nem1/Spo7 homologue in *B. dothidea*, and structural analysis showed that Nem1 contains a transmembrane domain and a characteristic HAD-like domain. Although the HAD-like domain is divergent in Nem1 homologues of different organisms, the phosphatase activity conferred by catalytic motif DXDX(T/V) is evolutionarily conserved (19, 40). Consistently, *B. dothidea* Nem1 possesses catalytic motif DLDET in the HAD-like domain, which is exactly the same as in yeast (30). Spo7 has two transmembrane domains and three other conserved regions. The split-ubiquitin membrane-based yeast two-hybrid and co-IP assays showed that Nem1 physically interacted with Spo7, and the CTR of Nem1 and the hydrophobicity of the LLI sequence in Spo7 are indispensable for their interaction.

To identify the catalytic substrate of Nem1/Spo7 complex, we first sought a candidate protein that interacts with Nem1 and Spo7. By performing affinity capture MS, co-IP, and yeast two-hybrid assays, we identified a phosphatidic acid phosphatase, Pah1, which physically interacted with Nem1/Spo7 complex by Spo7, and the acidic tail sequence of Pah1 was essential for the interaction with Spo7. Then the Phos-tag assay was performed, and the results showed that Pah1 was indeed dephosphorylated by Nem1/Spo7. Previous studies reported that dephosphorylated Pah1 catalyzes the conversion of PA to DAG, which is further converted to TAG for storage in LDs. In this study, we tested the TAG and LD accumulation patterns in single-deletion mutants of Nem1/Spo7-Pah1 cascade, and the results showed that both TAG contents and LD numbers were seriously decreased in the Δ*Nem1* and Δ*Spo7* mutants. Under the same conditions, the Δ*Pah1* mutant exhibited a more severe defect, which is consistent with their orthologues in yeast (41) and filamentous fungi (33). These results suggested that the role of Nem1/Spo7-Pah1 in regulating TAG and subsequent LD synthesis was evolutionarily conserved. In addition, both the Δ*Nem1* and Δ*Spo7* mutants of *B. dothidea* showed declined tolerance to the Demethylation inhibitor (DMI) fungicide tebuconazole (Fig. S9), which is widely used for the control of apple ring rot. Given that tebuconazole interferes with the synthesis of ergosterol, which is converted to sterol ester (SE) (14), another important component in LDs, we speculate that the response defects of the Δ*Nem1* and Δ*Spo7* mutants toward tebuconazole may be associated with their lipid abnormalities, and the underlying mechanism needs to be further studied.

Notably, we report for the first time that single depletion of Nem1/Spo7-Pah1 cascade resulted in abnormal increases in the size of protoplasts. In addition, all the three single-deletion mutants of Nem1/Spo7-Pah1 displayed abnormal nuclear membrane expansion, and the Δ*Pah1* mutant exhibited a more severe defect than the Δ*Nem1* and Δ*Spo7* phosphatase mutants. qRT-PCR and ChIP-qPCR assays revealed that dephosphorylated Pah1 upon Nem1/Spo7 could bind to the promoters of genes encoding key enzymes for nuclear membrane synthesis, which exhibited a significant upregulation in the Δ*Nem1*, Δ*Spo7*, and Δ*Pah1* mutants. Consistently, the increase in the mRNA levels was higher in the Δ*Pah1* mutant than in the Δ*Nem1* and Δ*Spo7* mutants. Moreover, knocking out of the most upregulated gene (*INO1*) repressed nuclear membrane expansion and restored a nearly normal spherical nucleus in most cells of all three deletion mutants. These results indicate that the dephosphorylated Pah1 functions as a transcriptional repressor of nuclear membrane synthesis genes to regulate nuclear envelope morphology. Interestingly, knockout of AnNem1 (Nem1 homologue in Aspergillus nidulans) led to small nuclei (42); in contrast, deletion of Nem1 in *B. dothidea* yielded expansive nuclei with a bigger size, suggesting the diverse functions and mechanisms of Pah1 proteins in regulating nuclear size of eukaryotes.

It is worth noting that Nem1 could not complement the stress response defects of yeast Nem1 deletion mutants. In addition, recent advances in the budding yeast and higher eukaryotes have been increasingly connecting LD dynamics to the regulation of autophagy, and TAG, as the key component in LDs, is demonstrated to be important for autophagy induction after nutrient starvation (43–45). Deletion of yeast Nem1, Spo7, or Pah1 seriously reduces TAG contents and LD numbers and finally affects the initiation of autophagy (26). However, in this study, our results showed that although the TAG and LD numbers were significantly decreased, autophagy occurred normally in the Δ*Nem1*, Δ*Spo7*, and Δ*Pah1* mutants (data not shown). These results indicate that the roles of the Nem1/Spo7-Pah1 cascade in regulating autophagy vary in different organisms despite their conserved functional domains.

In filamentous fungi, such as A. fumigatus, M. oryzae, and F. graminearum, Nem1 is involved in regulating mycelial growth and conidiation but not in conidial morphology or germination (32–35). Nevertheless, in this study, disruption of either Nem1 or Spo7 affected hyphal growth and conidiation as well as conidial morphology and germination. Moreover, F. graminearum Nem1/Spo7 (FgNem1/Spo7) complex plays important roles in regulation of pigment metabolism but not in response to cell wall-damaging stress mediated by Congo red or calcofluor white (CFW) in F. graminearum, while *B. dothidea* Nem1/Spo7 single-deletion mutants displayed normal pigment and hypersensitivity toward CR and CFW, which is similar to the case with in A. fumigatus. Moreover, A. fumigatus NemA (AfNemA) is involved in regulating responses to stress mediated by iron starvation, while the Δ*Nem1* and Δ*Spo7* mutants exhibited no defects in sensitivity toward deficiency of iron in *B. dothidea*. In addition, FgNem1/Spo7 complex is critical for full virulence of F. graminearum mainly by affecting mycotoxin deoxynivalenol (DON) production and response to plant immunity mediated by reactive oxygen. In contrast, knockout of M. oryzae Nem1 (MoNem1) does not affect the pathogenicity of M. oryzae despite its lower growth rate. In this study, our results showed that disruption of the *B. dothidea* Nem1/Spo7-Pah1 cascade significantly reduced the virulence of *B. dothidea* on both apple fruit and branch, and the defects in phytoalexin tolerance may contribute to the reduced virulence of the Δ*Nem1* and Δ*Spo7* mutants. We could not determine the sensitivity of the *B. dothidea* Δ*Pah1* mutant toward various environmental pressures due to its extremely declined hyphal growth rate and complete loss of asexual reproduction. Therefore, it is difficult to analyze the reasons causing the abolished virulence of the Δ*Pah1* mutant, but it is certain that growth defects are one of the important reasons for the decline of the pathogenicity. These results suggest that Nem1/Spo7 complex plays diverse roles in regulating development, secondary metabolism, stresses responses, and pathogenicity in different fungal species. Taken together, the results indicate that the phosphatase cascade Nem1/Spo7-Pah1 presents great functional differentiation among diverse species despite the existence of several conserved domains; it is speculated that there is a pattern of evolution in which different species adapt to survive, reproduce, and spread.

**FIG 9:**
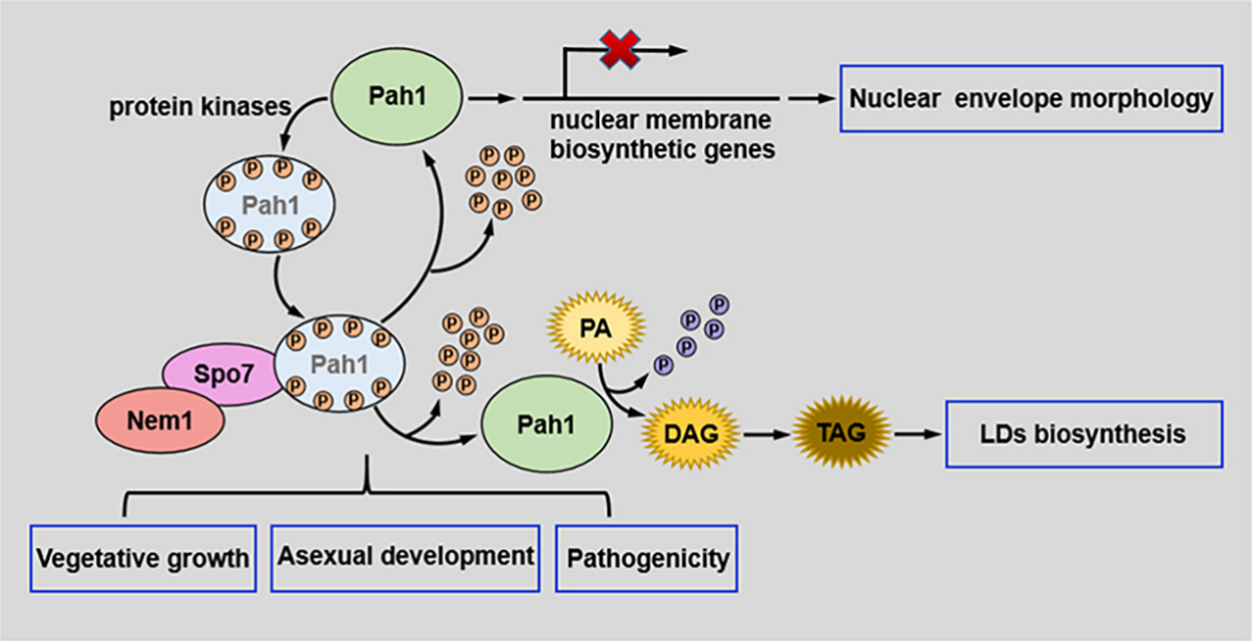
Proposed model for functions of Nem1/Spo7-Pah1 phosphatase cascade in *Botryosphaeria dothidea.* The phosphatidic acid (PA) phosphatase Pah1 is phosphorylated by multiple protein kinases, and then the phosphorylated Pah1 is dephosphorylated by protein phosphatase complex Nem1/Spo7. Subsequently, the dephosphorylated Pah1 catalyzes PA conversion to diacylglycerol (DAG) and subsequent TAG and LD synthesis. Moreover, dephosphorylated Pah1 could bind to the promoter of key nuclear membrane biosynthesis genes and functioned as a transcriptional repressor to regulate nuclear membrane morphology. In addition, the Nem1/Spo7-Pah1 cascade was involved in regulating hyphal growth, asexual development, and pathogenicity of *B. dothidea*.

In conclusion, our results indicate that protein phosphatase complex Nem1/Spo7 interacted with and dephosphorylated the PA phosphatase Pah1. Dephosphorylated Pah1 was active and promoted the conversion of PA to TAGs and subsequent LD synthesis. Moreover, Nem1/Spo7-dependently dephosphorylated Pah1 could bind to the promoters of key nuclear membrane biosynthesis genes and functioned as a transcriptional repressor to regulate nuclear membrane morphology. In addition, phenotypic analyses showed that the Nem1/Spo7-Pah1 cascade was involved in regulating hyphal growth, asexual development, stress responses, and pathogenicity of *B. dothidea* (Fig. 9). The results of this study will provide a theoretical basis for using the phosphatase cascade Nem1/Spo7-Pah1 as a target for phosphatase inhibitor or fungicide development and RNA interference (RNAi) for the management of apple ring rot, and they also provide insights on comprehensive functional analysis of phosphatase complex Nem1/Spo7 in fungi.

## MATERIALS AND METHODS

### Transcription profiling assays.

Total RNA was isolated using TRIzol reagent (TaKaRa Inc., Dalian, China) according to the manufacturer’s instructions. The sequencing libraries were generated using the NEBNext Ultra RNA library prep kit for Illumina (New England Biolabs [NEB], Ipswich, MA, USA) as described previously (46). The clustering of the index-coded samples was performed on a cBot cluster generation system using TruSeq cluster kit v3-cBot-HS (Illumina, Bologna, Italy) according to the manufacturer’s instructions. The library preparations were sequenced on an Illumina Nova platform, and 150-bp paired-end reads were generated. Differential expression analysis was performed using the DESeq2 R package (1.16.1).

### Affinity capture MS analysis.

Nem1-GFP and GFP-Spo7 were transferred into the LW03, and the resulting transformants were used for protein extraction as described previously (47). The supernatant protein was transferred into a sterilized Eppendorf tube. Anti-GFP agarose (40 μL; Chromo Tek, Munich, Germany) was added to capture Nem1-GFP or GFP-Spo7 interacting proteins following the manufacturer’s instructions. After incubation overnight, the agarose was washed three times with TBS (20 mM Tris-HCl, 500 mM NaCl [pH 7.5]). Proteins binding to the beads were boiled with 90 μL of TBS supplemented with 15 μL of 10% SDS. After centrifugation, proteins in the supernatant were digested with trypsin as described previously (47). Tryptic peptides were analyzed by mass spectrometry as previously described (48).

### Fungal strains and culture conditions.

*B. dothidea* strain LW03 (LXS030101) was single-spore isolated from infected red Fuji fruit showing typical symptoms of apple ring rot in Shandong Province and was used as the wild-type (WT) strain for transformation experiments throughout this study. Strains were grown on potato dextrose agar (PDA; 200 g of potato, 20 g of glucose, 10 g of agar and 1 L of water), minimal medium (MM; 0.5 g of KCl, 2 g of NaNO_3_, 1 g of KH_2_PO_4_, 0.5 g of MgSO_4_·7H_2_O, 0.01 g of FeSO_4_·7H_2_O, 30 g of sucrose, 200 μL of trace element, 15 g of agar, and 1 L of water [pH 6.9]), bark medium (100 g of bark from Fuji apple tree and 15 g of agar in 1 L of water), and fruit medium (200 g of Fuji apples and 15 g of agar in 1 L of water) for mycelial growth tests. For fruiting body and conidium induction, each fungal strain was grown on carrot agar medium (200 g of carrot and 15 g of agar in 1 L of water) with near-UV light (wavelength, 365 nm; HKiv, Xiamen, China). Mycelia used for protein extraction or fluorescence observation were grown in yeast extract-peptone-dextrose (YEPD) medium (10 g of peptone, 3 g of yeast extract, 20 g of d-glucose, made up to 1 L [pH 6.7]). For determination of fungal growth under phytoalexin stress conditions, mycelial plugs (5 mm in diameter) taken from the periphery of a 3-day-old colony of each strain were inoculated onto PDA amended or not with phytoalexin stress mediator *O*-aminophenol at concentrations indicated in the figure legends. After 3 days of incubation at 25°C, colony diameter in each plate was measured. Each experiment was repeated three times.

### Generation of deletion and complemented mutants.

Gene deletion vectors were constructed using a strategy based on double-joint PCR (49). Primers used to amplify the flanking sequences of each gene are listed in the Table S1 in the supplemental material. Briefly, the knockout constructs were transformed into wild-type strain LW03 protoplasts by employing polyethylene glycol (PEG)-mediated protoplast transformation (50). Putative gene deletion mutants were identified by PCR with relevant primers (Table S2).

To construct the Nem1-GFP fusion cassette, the *NEM1* fragment containing the native promoter and open reading frame (ORF; without stop codon) was amplified with primers P19 and P20 (Table S1) and the resulting PCR products were cotransformed with XhoI-digested pYF11 into yeast strain XK1-25 (51). The recombined *NEM1*-*GFP* fusion vector was constructed with the alkali-cation yeast transformation kit (MP Biomedicals, Solon, OH, USA). Subsequently, the Nem1-GFP fusion vector was recovered from the yeast transformants by using the yeast plasmid extract kit (Solarbio, Beijing, China) and then transferred into E. coli strain DH5α. The *GFP*-*SPO7*, *PAH1*-*GFP*, *PAH1*-*FLAG*, *SEC63*-*GFP*, and *NEM1-FLAG* fusion vectors were generated similarly. Finally, the *NEM1*-*GFP*, *GFP*-*SPO7*, and *PAH1*-*GFP* plasmids were introduced into the deletion mutants for generation of complemented strains. *SEC63*-*GFP* plasmid was transformed into LW03 and various deletion mutants to obtain fluorescence-labeled strains.

### Histochemical analysis of LDs and quantification of TAG production.

Nile red or boron-dipyrromethene (BODIPY) was used for lipid droplet (LD) staining. For Nile red staining, hyphae or conidia of each strain were incubated in Nile red staining solution (20 mg mL^−1^) of polyvinylpyrrolidone and 2.5 mg mL^−1^ of Nile red oxazone (Sigma-Aldrich, St. Louis, MO, USA) in 50 mM Tris-maleate buffer (pH 7.5) for a few seconds, and then LDs were examined under a microscope with an episcopic fluorescence attachment. For BODIPY staining, hyphae or conidia were stained for 10 min in the dark with 1 μM BODIPY493/503 (Thermo Fisher Scientific, MA, USA) dissolved in 0.1× phosphate-buffered saline (PBS) buffer. After washing twice with 1 × PBS buffer, LDs were examined with a confocal microscope (TCS SP5; Leica, Germany). Each experiment was repeated three times.

To determine TAG content, fresh hyphae were digested with cellulase (Kaiyang, Shanghai, China), lysozyme (Kaiyang) and Driselase (Sigma-Aldrich) for 4 h in 0.7 M NaCl to prepare for protoplasts. Then triglyceride quantification kit E1013 (Applygen, Beijing, China) was used to quantify the TAG concentration after resuspension of the 50 mg of protoplasts in 1 mL of lysis buffer according to the manufacturer’s instructions. The protein concentrations in the resulting lysis supernatant were determined by the bicinchoninic acid method with protein quantification kit P1511 (Applygen) as a reference. The experiment was repeated three times.

### Western blotting.

For total protein extractions, mycelia were ground in liquid nitrogen. Approximately 200 mg of finely ground mycelia powder was resuspended in 1 mL of extraction buffer (50 mM Tris-HCl [pH 7.5], 100 mM NaCl, 5 mM EDTA, 1% Triton X-100, 2 mM phenylmethylsulfonyl fluoride [PMSF]) and 10 μL of protease inhibitor cocktail (Sangon, Shanghai, China). After homogenization with a vortex shaker, the lysate was centrifuged. The supernatant was mixed with protein loading buffer and boiled for 5 min. The resulting proteins were separated by 12.5% sodium dodecyl sulfate-polyacrylamide gel electrophoresis (SDS-PAGE) and transferred to a polyvinylidene fluoride (PVDF) membrane. To test the expression pattern of Nem1, Spo7, and Pah1 in complemented strains, monoclonal anti-GFP antibody 32146 (Abcam, Cambridge, MA, USA) was used at a 1:100,000 dilution for immunoblot analyses. The samples were also detected with monoclonal anti-glyceraldehyde-3-phosphate dehydrogenase (anti-GAPDH) antibody EM1101 (Hangzhou HuaAn Biotechnology Co., Ltd.) as a reference. Incubation with a secondary antibody and chemiluminescent detection were performed as described previously (52). Each experiment was repeated three times.

### Co-IP assay.

The GFP and Flag fusion constructs were transformed in pairs into LW03. Transformants expressing the fusion constructs were confirmed by Western blotting. In addition, the transformants bearing a single fusion construct were used as references. For coimmunoprecipitation (co-IP) assays, total proteins were isolated and incubated with the anti-GFP agarose (Chromo Tek, Martinsried, Germany). Proteins eluted from agarose were analyzed by Western blotting detection with monoclonal anti-Flag antibody A9044 (Sigma-Aldrich, St. Louis, MO, USA) and monoclonal anti-GFP antibodies. Each protein sample was also detected with anti-GAPDH antibody as a reference. The experiment was repeated three times.

### Phos-tag assay.

After 10 5-mm mycelial plugs of each strain were cultivated in YEPD medium at 25°C for 42 h, hyphae of each strain were harvested for protein extraction. The resulting protein samples were resolved on 8% SDS-polyacrylamide gels prepared with 25 μM Phos-tag binding reagent acrylamide (APE×BIO, F4002) and 100 μM MnCl_2_ (the separating gel included 1.33 mL of acrylamide/bis solution [30%, 29:1, MA0071; Meilune Biotechnology, Dalian, China], 1.25 mL of bis-Tris [1.4 M, pH 6.8, B9754; Sigma-Aldrich, St. Louis, MO, USA], 2.31 mL of H_2_O, 25 μL of Phos-tag [5 mM], 50 μL of MnCl_2_ [10 mM], 25 μL of ammonium persulfate [APS; 10%], and 5 μL of *N*,*N*,*N*′,*N*′-tetramethylethylenediamine [TEMED]; the stacking gel included 0.3 mL of acrylamide/bis solution, 0.5 mL of bis-Tris, 1.19 mL of H_2_O, 10 μL of APS, and 2 μL of TEMED). Gels were electrophoresed at 20 mA/gel for 4 h. Prior to protein transfer, gels were first equilibrated three times in transfer buffer containing 5 mM EDTA for 5 min and further equilibrated in transfer buffer without EDTA for 5 min two times. Protein transform from the Mn^2+^-Phos-tag acrylamide gel to the PVDF membrane was performed for 5 h at 100 V at 0°C, and finally, the membrane was analyzed by Western blotting with the anti-Flag antibody. Each protein sample was also separated by 12.5% SDS-PAGE and detected with anti-Flag antibody as a reference. The experiment was repeated three times.

### Split-ubiquitin membrane-based yeast two-hybrid assay.

In order to construct plasmids for the split-ubiquitin membrane-based yeast two-hybrid assay, coding sequence of each tested gene was amplified from cDNA of LW03 with the primer pairs indicated in Table S1. The wild-type or mutated form of the *NEM1*, *SPO7*, or *PAH1* cDNA fragment was fused to the C-terminal or N-terminal half of ubiquitin (Cub) and the artificial transcription factor LexA-VP16 (Dualsystems Biotech, Zurich City, Switzerland). In addition, *NEM1* or *PAH1* was fused to the mutated C-terminal or N-terminal half of ubiquitin (Nub) (Dualsystems Biotech, Switzerland). The pairs of yeast two-hybrid plasmids were cotransformed into S. cerevisiae strain NMY51 following the LiAc/single-stranded DNA/PEG transformation protocol (53). Transformants were grown at 30°C for 3 days on synthetic medium lacking leucine (Leu) and tryptophan (Trp) and then transferred to medium stripped of Trp, Leu, histidine (His), and adenine (Ade) to assess binding activity. The experiments were repeated three times.

### qRT-PCR analysis.

For total RNA extraction, the harvested mycelia of each strain were ground in liquid nitrogen, total RNA was then isolated from mycelia of each sample with TaKaRa RNAiso reagent (TaKaRa Biotechnology Co., Dalian, China), and 10 mg of each RNA sample was used for reverse transcription with a RevertAid H Minus first-strand cDNA synthesis kit (Fermentas Life Sciences, Burlington, ON, Canada). The expression levels of *INO1*, *INO2*, and *OPI3* were determined by quantitative real-time PCR (qRT-PCR) using the primers listed in Table S1. For each sample, the *ACTIN* gene was measured as an internal control using qPCR (0.4 μL of primer-F, 0.4 μL of primer-R, 10 μL of SYBR [TaKaRa Biotechnology Co., Dalian, China], 1 μL of cDNA and 8.2 μL of H_2_O) and an RT-PCR program (initial denaturation at 95°C for 3 min, followed by 40 cycles of denaturation at 95°C for 20 s, annealing at 53°C for 25 s, extension at 72°C for 30 s, and final extension at 72°C for 10 min). The experiment was repeated three times independently.

### ChIP assays.

Chromatin immunoprecipitation (ChIP) was performed as described previously (54). Briefly, mycelia were cross-linked with 1% formaldehyde for 10 min and then stopped with 125 mM glycine. Subsequently, samples were grounded in liquid nitrogen and suspended in lysis buffer with protease inhibitor. DNA was sheared into 200- to 500-bp fragments in a Bioruptor Plus (UCD-300; Liege, Belgium). After centrifugation, the supernatant was diluted with 10× ChIP dilution buffer. The DNA fragments’ supernatants were incubated with the GFP antibody 290 (Abcam, Cambridge, MA, USA) as the IP sample or incubated with anti-IgG1 antibody MA1-10406 (Invitrogen-Thermo Fisher Scientific, MA, USA) as the mock sample. In addition, protein A agarose beads (sc-2001; Santa Cruz Biotechnology, Dallas, TX, USA) were added into IP and mock samples before incubation with antibodies to remove the background. The beads were washed after a second addition for immunoprecipitation, and the immunoprecipitated complexes were then eluted from beads. Then 5 M NaCl and 2 μL of RNase were added into ChIP and mock samples to reverse crossing-linking and digested with proteinase K. Next, an equal volume of phenol-chloroform-isoamyl alcohol was added into each sample to precipitate DNA. Finally, the IP or mock DNA samples were quantified by qPCR assays with primers listed in Table S2. Relative enrichment levels were determined using the fold enrichment method. The experiment was repeated three times.

### Determination of asexual reproduction and pathogenicity assays.

To detect asexual development, aerial hyphae of 4-day-old cultures of LW03, Δ*Nem1*, Δ*Spo7*, and complemented strains and 7-day-old cultures of Δ*Pah1* grown on carrot agar plates were pressed down with 500 μL of 0.25% Tween 60 and incubated under black light (wavelength, 365 nm; HKiv Co., Ltd., Xiamen, China); fruiting body formation and conidium production were assayed after incubation at 25°C for 2 weeks.

The pathogenicity of *B. dothidea* was analyzed on young Fuji apple fruits and 1-year-old branches. To test the virulence of each strain on wounded apple fruits, holes were punched into the apple skin (5 mm in diameter, 3 mm in depth), and mycelial plugs (5 mm in diameter) of each strain were taken from the edge of a 3-day-old colony grown on PDA and then transferred into the holes of the apple fruits. For pathogenicity tests on branches, the phloem was exposed after removal of 5-mm-diameter samples of epidermis of branches, and then the mycelial plugs of each strain were transferred onto the exposed phloem. In addition, water agar plugs without mycelia were used as negative controls. The inoculated fruits and branches were placed under high-humidity conditions (95%) at 25°C for 7 days and 10 days, respectively. These experiments were repeated three times independently, and 20 samples were used for each strain each time.

### Data availability.

The RNA sequencing data set is available in supplemental material.

## References

[1] Marsberg A, Kemler M, Jami F, Nagel JH, Postma-Smidt A, Naidoo S, Wingfield MJ, Crous PW, Spatafora JW, Hesse CN, Robbertse B, Slippers B. 2017. Botryosphaeria dothidea: a latent pathogen of global importance to woody plant health. Mol Plant Pathol 18:477–488. doi:10.1111/mpp.12495. 27682468 PMC6638292

[2] Tang W, Ding Z, Zhou Z, Wang Y, Guo L. 2012. Phylogenetic and pathogenic analyses show that the causal agent of apple ring rot in China is Botryosphaeria dothidea. Plant Dis 96:486–496. doi:10.1094/PDIS-08-11-0635. 30727432

[3] Xue D-S, Liu J, Li B-H, Xu X-M, Liu N, Lian S, Dong X-L, Wang C-X. 2021. Effect of rainfall and temperature on perithecium production of Botryosphaeria dothidea on cankered apple branches. Phytopathology 111:982–989. doi:10.1094/PHYTO-07-20-0262-R. 33210989

[4] Jurick WM, Vico I, Gaskins VL, Janisiewicz WJ, Peter KA. 2013. First report of Botryosphaeria dothidea causing white rot on apple fruit in Maryland. Plant Dis 97:999. doi:10.1094/PDIS-01-13-0053-PDN. 30722551

[5] Ma Z, Luo Y, Michailides TJ. 2001. Resistance of Botryosphaeria dothidea from pistachio to iprodione. Plant Dis 85:183–188. doi:10.1094/PDIS.2001.85.2.183. 30831940

[6] Wang Y, Zhang W, Liu B, Luan B, Wang P. 2010. Research on resistance and geographical distribution of Botryosphaeria dothidea from apple to tebuconazole in Shandong Province. J Fruit Sci 27:961–964.

[7] Wang L, Tu H, Hou H, Zhou Z, Yuan H, Luo C, Gu Q. 2022. Occurrence and detection of carbendazim resistance in Botryosphaeria dothidea from apple orchards in China. Plant Dis 106:207–214. doi:10.1094/PDIS-06-20-1204-RE. 34227835

[8] Cohen P. 2000. The regulation of protein function by multisite phosphorylation—a 25 year update. Trends Biochem Sci 25:596–601. doi:10.1016/s0968-0004(00)01712-6. 11116185

[9] Alonso A, Sasin J, Bottini N, Friedberg I, Friedberg I, Osterman A, Godzik A, Hunter T, Dixon J, Mustelin T. 2004. Protein tyrosine phosphatases in the human genome. Cell 117:699–711. doi:10.1016/j.cell.2004.05.018. 15186772

[10] Gohla A, Birkenfeld J, Bokoch GM. 2005. Chronophin, a novel HAD-type serine protein phosphatase, regulates cofilin-dependent actin dynamics. Nat Cell Biol 7:21–29. doi:10.1038/ncb1201. 15580268

[11] Goldberg J, Huang HB, Kwon YG, Greengard P, Nairn AC, Kuriyan J. 1995. Three-dimensional structure of the catalytic subunit of protein serine/threonine phosphatase-1. Nature 376:745–753. doi:10.1038/376745a0. 7651533

[12] Khondker S, Han GS, Carman GM. 2022. Phosphorylation-mediated regulation of the Nem1-Spo7/Pah1 phosphatase cascade in yeast lipid synthesis. Adv Biol Regul 84:100889. doi:10.1016/j.jbior.2022.100889. 35231723 PMC9149063

[13] Kwiatek JM, Han GS, Carman GM. 2020. Phosphatidate-mediated regulation of lipid synthesis at the nuclear/endoplasmic reticulum membrane. Biochim Biophys Acta Mol Cell Biol Lipids 1865:158434. doi:10.1016/j.bbalip.2019.03.006. 30910690 PMC6755077

[14] Wang CW. 2015. Lipid droplet dynamics in budding yeast. Cell Mol Life Sci 72:2677–2695. doi:10.1007/s00018-015-1903-5. 25894691 PMC11113813

[15] Karanasios E, Han GS, Xu Z, Carman GM, Siniossoglou S. 2010. A phosphorylation-regulated amphipathic helix controls the membrane translocation and function of the yeast phosphatidate phosphatase. Proc Natl Acad Sci USA 107:17539–17544. doi:10.1073/pnas.1007974107. 20876142 PMC2955120

[16] Karanasios E, Barbosa AD, Sembongi H, Mari M, Han G-S, Reggiori F, Carman GM, Siniossoglou S. 2013. Regulation of lipid droplet and membrane biogenesis by the acidic tail of the phosphatidate phosphatase Pah1p. Mol Biol Cell 24:2124–2133. doi:10.1091/mbc.E13-01-0021. 23657815 PMC3694796

[17] O’Hara L, Han GS, Peak-Chew S, Grimsey N, Carman GM, Siniossoglou S. 2006. Control of phospholipid synthesis by phosphorylation of the yeast lipin Pah1p/Smp2p Mg^2+^-dependent phosphatidate phosphatase. J Biol Chem 281:34537–34548. doi:10.1074/jbc.M606654200. 16968695 PMC1769310

[18] Santos-Rosa H, Leung J, Grimsey N, Peak-Chew S, Siniossoglou S. 2005. The yeast lipin Smp2 couples phospholipid biosynthesis to nuclear membrane growth. EMBO J 24:1931–1941. doi:10.1038/sj.emboj.7600672. 15889145 PMC1142606

[19] Siniossoglou S, Santos-Rosa H, Rappsilber J, Mann M, Hurt E. 1998. A novel complex of membrane proteins required for formation of a spherical nucleus. EMBO J 17:6449–6464. doi:10.1093/emboj/17.22.6449. 9822591 PMC1170993

[20] Choi H-S, Su W-M, Morgan JM, Han G-S, Xu Z, Karanasios E, Siniossoglou S, Carman GM. 2011. Phosphorylation of phosphatidate phosphatase regulates its membrane association and physiological functions in Saccharomyces cerevisiae: identification of Ser^602^, Thr^723^, and Ser^744^ as the sites phosphorylated by CDC28 (CDK1)-encoded cyclin-dependent kinase. J Biol Chem 286:1486–1498. doi:10.1074/jbc.M110.155598. 21081492 PMC3020757

[21] Choi HS, Su WM, Han GS, Plote D, Xu Z, Carman GM. 2012. Pho85p-Pho80p phosphorylation of yeast Pah1p phosphatidate phosphatase regulates its activity, location, abundance, and function in lipid metabolism. J Biol Chem 287:11290–11301. doi:10.1074/jbc.M112.346023. 22334681 PMC3322823

[22] Pascual F, Hsieh LS, Soto-Cardalda A, Carman GM. 2014. Yeast Pah1p phosphatidate phosphatase is regulated by proteasome-mediated degradation. J Biol Chem 289:9811–9822. doi:10.1074/jbc.M114.550103. 24563465 PMC3975026

[23] Su WM, Han GS, Casciano J, Carman GM. 2012. Protein kinase A-mediated phosphorylation of Pah1p phosphatidate phosphatase functions in conjunction with the Pho85p-Pho80p and Cdc28p-cyclin B kinases to regulate lipid synthesis in yeast. J Biol Chem 287:33364–33376. doi:10.1074/jbc.M112.402339. 22865862 PMC3460439

[24] Su WM, Han GS, Carman GM. 2014. Cross-talk phosphorylations by protein kinase C and Pho85p-Pho80p protein kinase regulate Pah1p phosphatidate phosphatase abundance in Saccharomyces cerevisiae. J Biol Chem 289:18818–18830. doi:10.1074/jbc.M114.581462. 24876385 PMC4081924

[25] Carman GM, Han GS. 2011. Regulation of phospholipid synthesis in the yeast Saccharomyces cerevisiae. Annu Rev Biochem 80:859–883. doi:10.1146/annurev-biochem-060409-092229. 21275641 PMC3565220

[26] Rahman MA, Mostofa MG, Ushimaru T. 2018. The Nem1/Spo7-Pah1/lipin axis is required for autophagy induction after TORC1 inactivation. FEBS J 285:1840–1860. doi:10.1111/febs.14448. 29604183

[27] Xu X, Okamoto K. 2018. The Nem1-Spo7 protein phosphatase complex is required for efficient mitophagy in yeast. Biochem Biophys Res Commun 496:51–57. doi:10.1016/j.bbrc.2017.12.163. 29305265

[28] Jin J-H, Lee K-T, Hong J, Lee D, Jang E-H, Kim J-Y, Lee Y, Lee S-H, So Y-S, Jung K-W, Lee D-G, Jeong E, Lee M, Jang Y-B, Choi Y, Lee MH, Kim J-S, Yu S-R, Choi J-T, La J-W, Choi H, Kim S-W, Seo KJ, Lee Y, Thak EJ, Choi J, Averette AF, Lee Y-H, Heitman J, Kang HA, Cheong E, Bahn Y-S. 2020. Genome-wide functional analysis of phosphatases in the pathogenic fungus Cryptococcus neoformans. Nat Commun 11:4212. doi:10.1038/s41467-020-18028-0. 32839469 PMC7445287

[29] Shukla S, Pillai AN, Rahaman A. 2018. A putative NEM1 homologue regulates lipid droplet biogenesis via PAH1 in Tetrahymena thermophila. J Biosci 43:693–706. doi:10.1007/s12038-018-9794-x. 30207315

[30] Han S, Bahmanyar S, Zhang P, Grishin N, Oegema K, Crooke R, Graham M, Reue K, Dixon JE, Goodman JM. 2012. Nuclear envelope phosphatase 1-regulatory subunit 1 (formerly TMEM188) is the metazoan Spo7p ortholog and functions in the lipin activation pathway. J Biol Chem 287:3123–3137. doi:10.1074/jbc.M111.324350. 22134922 PMC3283218

[31] Satow R, Chan TC, Asashima M. 2002. Molecular cloning and characterization of dullard: a novel gene required for neural development. Biochem Biophys Res Commun 295:85–91. doi:10.1016/S0006-291X(02)00641-1. 12083771

[32] Winkelströter LK, Dolan SK, Fernanda Dos Reis T, Bom VLP, Alves de Castro P, Hagiwara D, Alowni R, Jones GW, Doyle S, Brown NA, Goldman GH. 2015. Systematic global analysis of genes encoding protein phosphatases in Aspergillus fumigatus. G3 (Bethesda) 5:1525–1539. doi:10.1534/g3.115.016766. 25943523 PMC4502386

[33] Liu N, Yun Y, Yin Y, Hahn M, Ma Z, Chen Y. 2019. Lipid droplet biogenesis regulated by the FgNem1/Spo7-FgPah1 phosphatase cascade plays critical roles in fungal development and virulence in Fusarium graminearum. New Phytol 223:412–429. doi:10.1111/nph.15748. 30767239

[34] Yun Y, Liu Z, Yin Y, Jiang J, Chen Y, Xu J-R, Ma Z. 2015. Functional analysis of the Fusarium graminearum phosphatome. New Phytol 207:119–134. doi:10.1111/nph.13374. 25758923

[35] Wang Y, Jiao T, Liu X, Lin F, Wu W. 2011. Functional characterization of a NEM1-like gene in Magnaporthe oryzae. Agric Sci China 10:1385–1390. doi:10.1016/S1671-2927(11)60131-4.

[36] Carman GM, Han GS. 2019. Fat-regulating phosphatidic acid phosphatase: a review of its roles and regulation in lipid homeostasis. J Lipid Res 60:2–6. doi:10.1194/jlr.S087452. 30530634 PMC6314256

[37] Kettle AJ, Batley J, Benfield AH, Manners JM, Kazan K, Gardiner DM. 2015. Degradation of the benzoxazolinone class of phytoalexins is important for virulence of Fusarium pseudograminearum towards wheat. Mol Plant Pathol 16:946–962. doi:10.1111/mpp.12250. 25727347 PMC6638480

[38] Pascual F, Soto-Cardalda A, Carman GM. 2013. PAH1-encoded phosphatidate phosphatase plays a role in the growth phase- and inositol-mediated regulation of lipid synthesis in Saccharomyces cerevisiae. J Biol Chem 288:35781–35792. doi:10.1074/jbc.M113.525766. 24196957 PMC3861629

[39] Rahman MA, Terasawa M, Mostofa MG, Ushimaru T. 2018. The TORC1-Nem1/Spo7-Pah1/lipin axis regulates microautophagy induction in budding yeast. Biochem Biophys Res Commun 504:505–512. doi:10.1016/j.bbrc.2018.09.011. 30201264

[40] Madera M, Vogel C, Kummerfeld SK, Chothia C, Gough J. 2004. The SUPERFAMILY database in 2004: additions and improvements. Nucleic Acids Res 32:D235–D239. doi:10.1093/nar/gkh117. 14681402 PMC308851

[41] Dubots E, Cottier S, Péli-Gulli M-P, Jaquenoud M, Bontron S, Schneiter R, De Virgilio C. 2014. TORC1 regulates Pah1 phosphatidate phosphatase activity via the Nem1/Spo7 protein phosphatase complex. PLoS One 9:e104194. doi:10.1371/journal.pone.0104194. 25117580 PMC4130541

[42] Son S, Osmani SA. 2009. Analysis of all protein phosphatase genes in Aspergillus nidulans identifies a new mitotic regulator, fcp1. Eukaryot Cell 8:573–585. doi:10.1128/EC.00346-08. 19181872 PMC2669206

[43] Dupont N, Chauhan S, Arko-Mensah J, Castillo EF, Masedunskas A, Weigert R, Robenek H, Proikas-Cezanne T, Deretic V. 2014. Neutral lipid stores and lipase PNPLA5 contribute to autophagosome biogenesis. Curr Biol 24:609–620. doi:10.1016/j.cub.2014.02.008. 24613307 PMC4016984

[44] Schepers J, Behl C. 2021. Lipid droplets and autophagy—links and regulations from yeast to humans. J Cell Biochem 122:602–611. doi:10.1002/jcb.29889. 33522032

[45] Shpilka T, Welter E, Borovsky N, Amar N, Mari M, Reggiori F, Elazar Z. 2015. Lipid droplets and their component triglycerides and steryl esters regulate autophagosome biogenesis. EMBO J 34:2117–2131. doi:10.15252/embj.201490315. 26162625 PMC4557665

[46] Liu N, Zhu M, Zhang Y, Wang Z, Li B, Ren W. 2022. Involvement of the autophagy protein Atg1 in development and virulence in Botryosphaeria dothidea. J Fungi 8:904. doi:10.3390/jof8090904. PMC9501979 36135629

[47] Gu Q, Chen Y, Liu Y, Zhang C, Ma Z. 2015. The transmembrane protein FgSho1 regulates fungal development and pathogenicity via the MAPK module Ste50-Ste11-Ste7 in Fusarium graminearum. New Phytol 206:315–328. doi:10.1111/nph.13158. 25388878

[48] Ding S-L, Liu W, Iliuk A, Ribot C, Vallet J, Tao A, Wang Y, Lebrun M-H, Xu J-R. 2010. The tig1 histone deacetylase complex regulates infectious growth in the rice blast fungus Magnaporthe oryzae. Plant Cell 22:2495–2508. doi:10.1105/tpc.110.074302. 20675574 PMC2929099

[49] Yu F, Gu Q, Yun Y, Yin Y, Xu J-R, Shim W-B, Ma Z. 2014. The TOR signaling pathway regulates vegetative development and virulence in Fusarium graminearum. New Phytol 203:219–232. doi:10.1111/nph.12776. 24684168

[50] Proctor RH, Hohn TM, McCormick SP. 1995. Reduced virulence of Gibberella zeae caused by disruption of a trichothecene toxin biosynthetic gene. Mol Plant Microbe Interact 8:593–601. doi:10.1094/mpmi-8-0593. 8589414

[51] Bruno KS, Tenjo F, Li L, Hamer JE, Xu JR. 2004. Cellular localization and role of kinase activity of PMK1 in Magnaporthe grisea. Eukaryot Cell 3:1525–1532. doi:10.1128/EC.3.6.1525-1532.2004. 15590826 PMC539019

[52] Yang Q, Yan L, Gu Q, Ma Z. 2012. The mitogen-activated protein kinase kinase kinase BcOs4 is required for vegetative differentiation and pathogenicity in Botrytis cinerea. Appl Microbiol Biotechnol 96:481–492. doi:10.1007/s00253-012-4029-9. 22526788

[53] Schiestl RH, Gietz RD. 1989. High efficiency transformation of intact yeast cells using single stranded nucleic acids as a carrier. Curr Genet 16:339–346. doi:10.1007/BF00340712. 2692852

[54] Ma T, Zhang L, Wang M, Li Y, Jian Y, Wu L, Kistler HC, Ma Z, Yin Y. 2021. Plant defense compound triggers mycotoxin synthesis by regulating H2B ub1 and H3K4 me2/3 deposition. New Phytol 232:2106–2123. doi:10.1111/nph.17718. 34480757 PMC9293436

