# Supplementary material for: The Phosphatase Cascade Nem1/Spo7-Pah1 Regulates Fungal Development, Lipid Homeostasis, and Virulence in Botryosphaeria dothidea
Source: Microbiol Spectr. 2023 May 16;11(3):e03881-22. doi: 10.1128/spectrum.03881-22 (PMC10269782; doi:10.1128/spectrum.03881-22)
Supplement: Supplemental file 1 — Supplemental material. Download spectrum.03881-22-s0001.pdf, PDF file, 1.2 MB [file spectrum.03881-22-s0001.pdf]

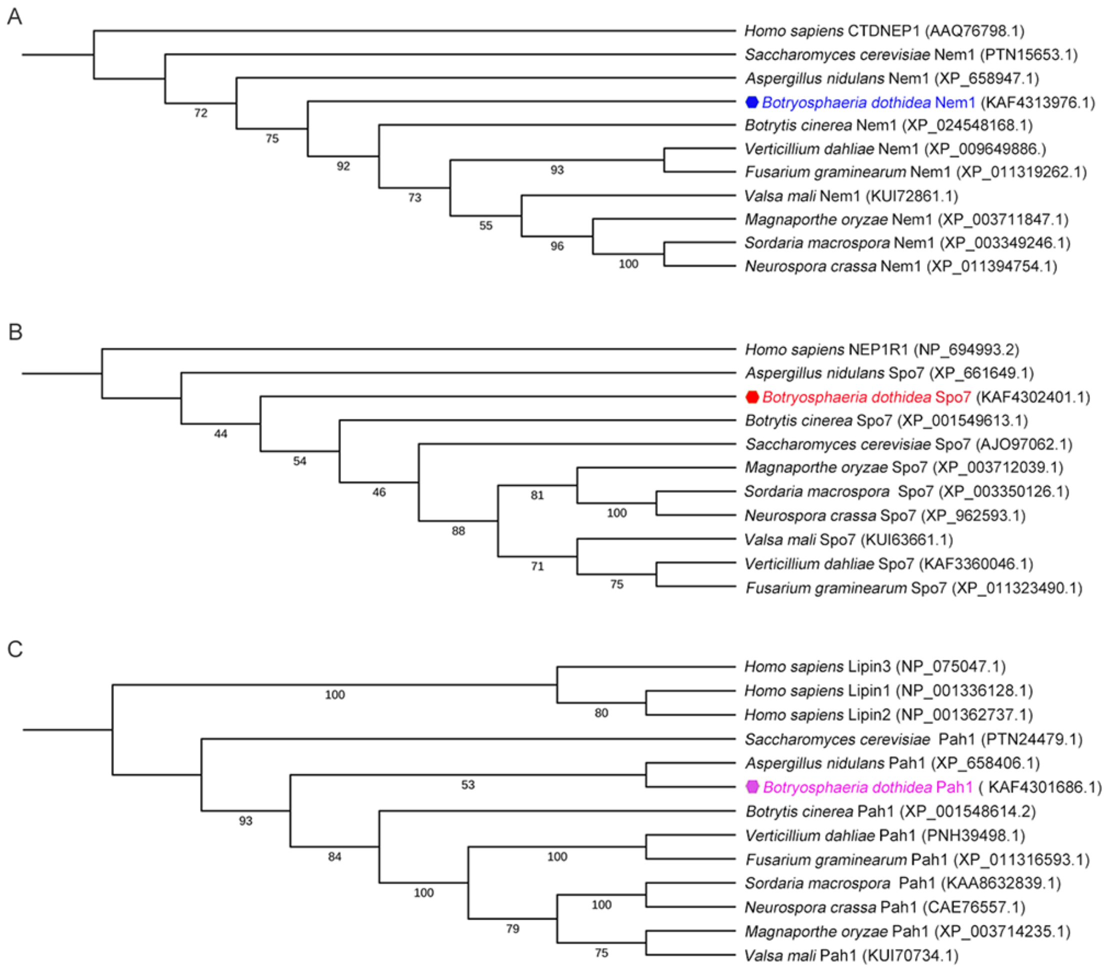

Fig. S1. Phylogenetic analysis of Nem1(A), Spo7 (B) and Pah1 (C) orthologs from *Botryosphaeria dothidea* and other organisms, including *Saccharomyces cerevisiae*, *Botrytis cinerea*, *Fusarium graminearum*, *Magnaporthe oryzae*, *Neurospora crassa*, *Aspergillus nidulans*, *Sordaria macrospora*, *Valsa mali*, *Verticillium dahliae* and Homo sapiens. The phylogenetic tree was constructed using IQTREE with the iTOL (Interactive Tree Of Life) analysis method.

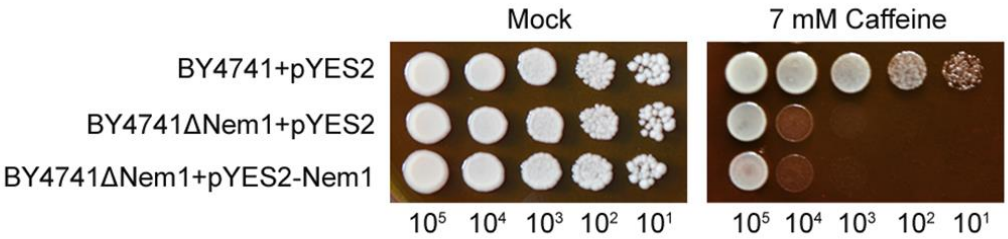

Fig. S2. *B. dothidea* Nem1 could not complement the growth defect of yeast Nem1 deletion mutant towards caffeine. The BY4741-derived deletion yeast mutant BY4741ΔNem1 was transformed with vector (pYES2) or *B. dothidea* Nem1 (pYES2-Nem1). Yeast cells including BY4741 + pYES2, BY4741ΔNem1 + pYES2 or BY4741ΔNem1+ pYES2-Nem1 were spotted on YPRG medium containing 7 mM caffeine or not (Mock). Photos were taken after 3 days of incubation at 30°C.

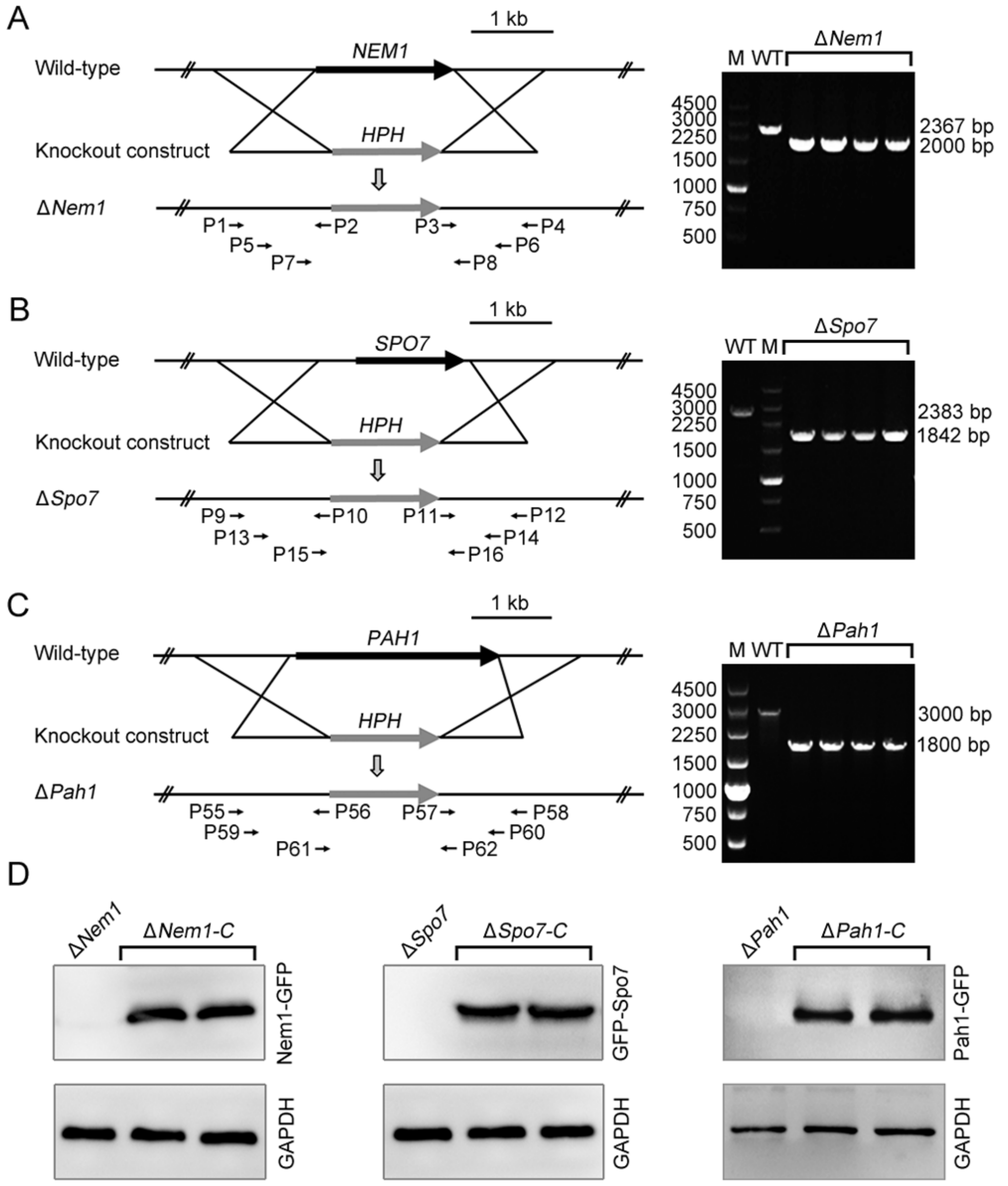

Fig. S3. Schematic representation of gene disruption strategy and identification of deletion and complemented mutants. (A) Schematic representation of the *NEM1* disruption strategy, the hygromycin resistance cassette (*HPH*) is denoted by the gray arrow (left panel). PCR identification of *NEM1* deletion mutants (right panel). (B) *SPO7* disruption strategy and PCR verification of the *SPO7* deletion mutant strain. (C) *PAH1* disruption strategy and PCR verification of the *PAH1* deletion mutant strain. (D) Western blotting analysis of expression patterns of fusion protein Nem1-GFP, GFP-Spo7 or Pah1-GFP.

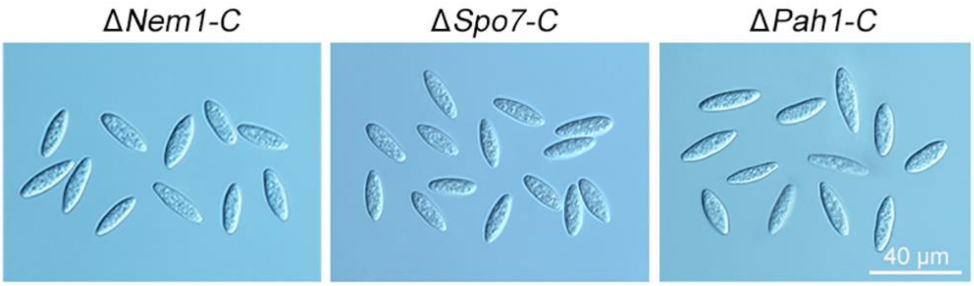

Fig. S4. Conidial morphology of each complemented strain.

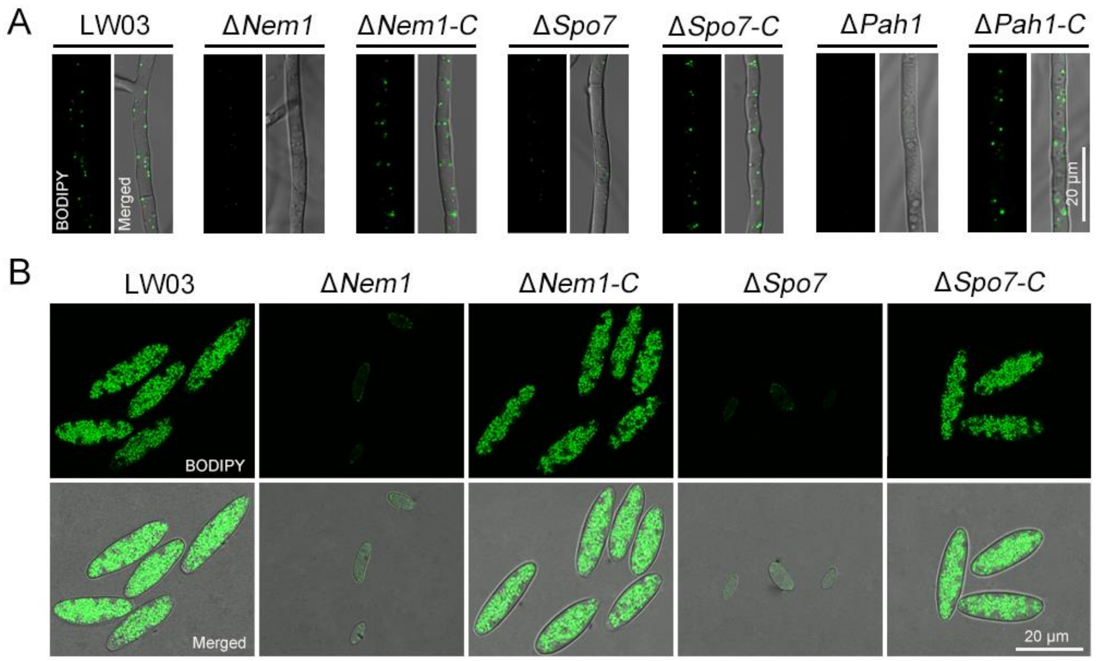

Fig. S5. LDs accumulation patterns in hyphae or conidia of each strain. Lipid droplets (LDs) within hyphae (A) or conidia (B) were stained with BODIPY (boron-dipyrromethene) and examined under a laser scanning confocal microscope.

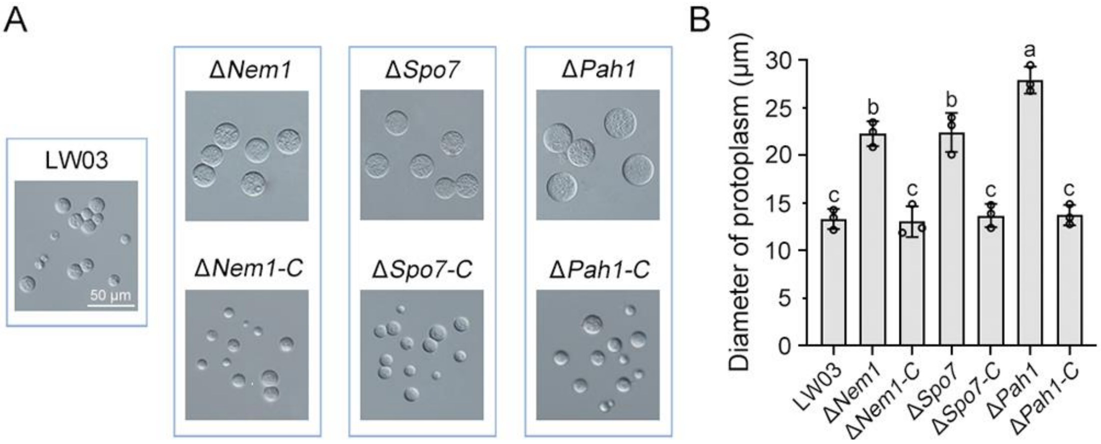

Fig. S6. Morphology of protoplasts produced by each strain. (A) Fresh mycelia (0.5g) of each strain were digested with 0.25g cellulase, 0.25g lysozyme and 0.1g driselase in 10ml 0.7M NaCl at 30℃ in a shaker with 85rpm for 3h, then the released protoplasts were observed under microscopy. (B) The size of protoplasts produced by each strain was quantified. Error bars represent standard errors of three replicated experiments and values on the bars followed by the same letter are not significantly different according to LSD test at *P*=0.05.

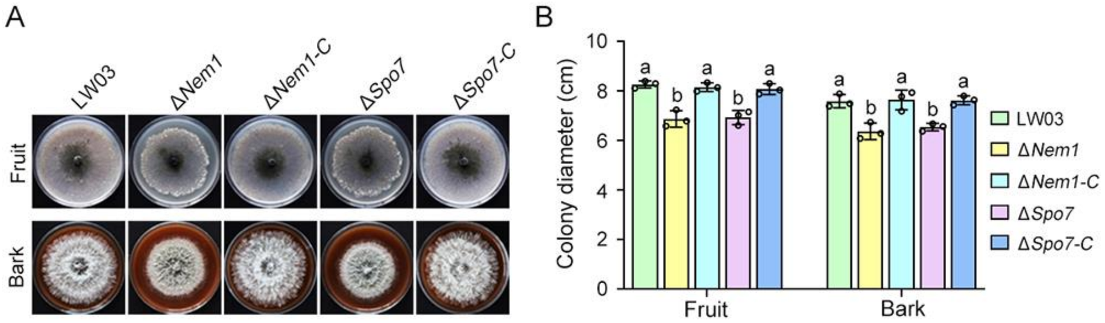

Fig. S7. The growth pattern of each strain on fruit and bark media. (A) Colony morphology of each strain grown on fruit and bark media for 2 and 4 days, respectively. (B) Colony diameter of each strain on fruit and bark media. Error bars represent standard errors of three replicated experiments and values on the bars followed by the same letter are not significantly different according to LSD test at *P*=0.05.

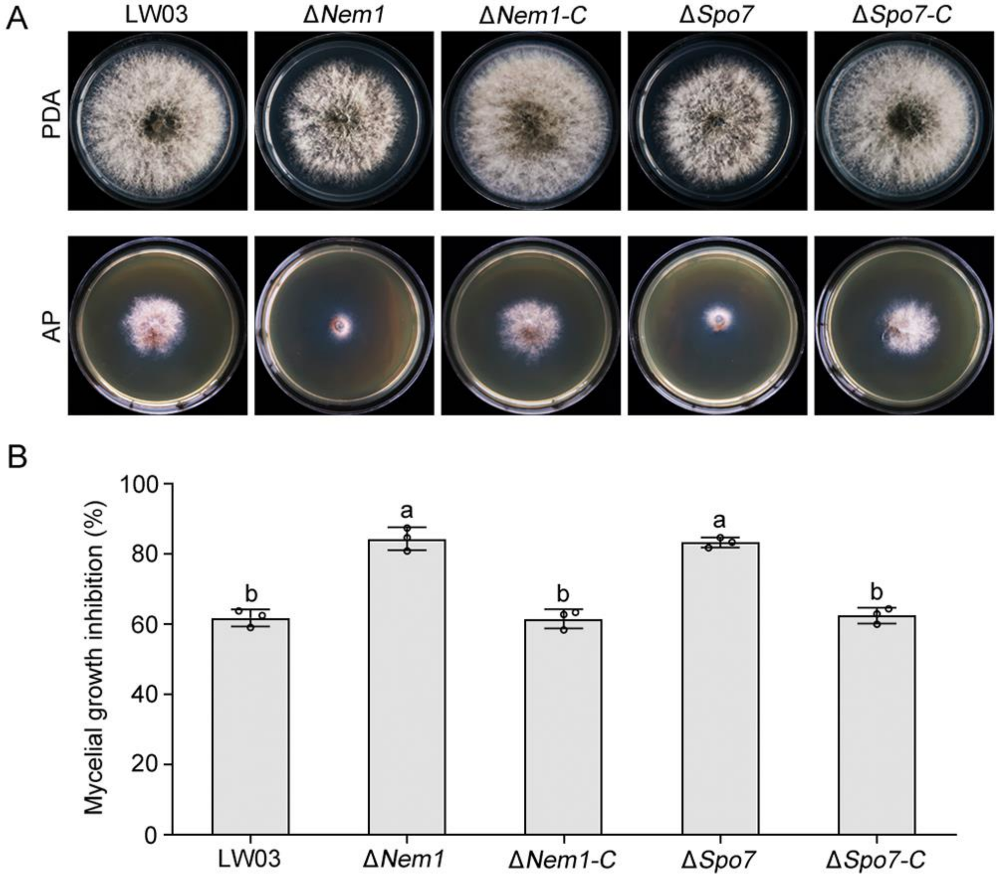

Fig. S8. Single deletion mutants of Nem1/Spo7 complex showed increased sensitivity to phytoalexin. (A) The wild-type strain LW03, Δ*Nem1*, Δ*Spo7*, Δ*Nem1-C* and Δ*Spo7-C* were grown on PDA with 0.04 mg/ml O-Aminophenol (AP) for 3 days. (B) Mycelial growth inhibition of each strain on PDA with 0.04 mg/ml AP for 3 days. Error bars represent standard errors of three replicated experiments and values on the bars followed by the same letter are not significantly different according to LSD test at *P*=0.05.

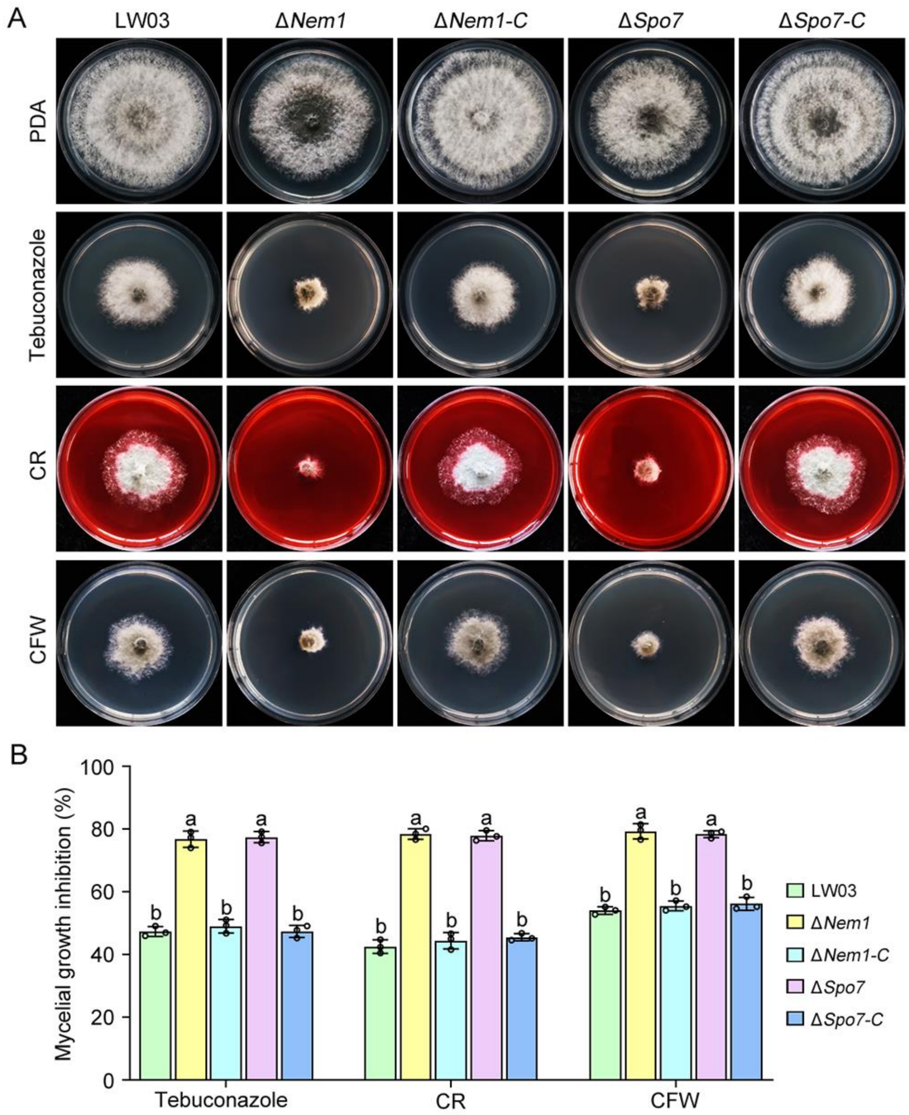

Fig. S9. Nem1/Spo7 complex regulates the response to multiple stressors. (A) Sensitivity of the LW03, Δ*Nem1*, Δ*Spo7* and complemented mutants to cell wall and fungicide stress. Comparisons were made on PDA amended without or with 0.8 g/L Congo red (CR), or 0.1 g/L Calcofluor white (CFW), or 0.125 μg/mL tebuconazole. All the plates were incubated at 25℃ for 2 days. (B) Mycelial growth inhibition in comparison with non-treatment was examined after each strain was incubated for 3 days on PDA amended with 0.8 g/L CR or 0.1 g/L CFW or 0.125 μg/mL tebuconazole. Error bars represent standard errors of three replicated experiments and values on the bars followed by the same letter are not significantly different according to LSD test at *P*=0.05

Table S1. A list of partial Nem1 and Spo7 co-interacting proteins identified by affinity capture in coupling with mass spectrometry.

Proteins	Gene description	Homologs in *Saccharomyces cerevisiae*
KAF4313976.1	Catalytic subunit of Nem1p-Spo7p phosphatase holoenzyme	Nem1
KAF4302401.1	Regulatory subunit of Nem1p-Spo7p phosphatase holoenzyme	Spo7
KAF4301686.1	Mg^{2+}- dependent phosphatidate (PA) phosphatase	Pah1
KAF4300968.1	Component of the CCR4-NOT transcriptional complex	Ccr4
KAF4306208.1	Nuclear SAM-dependent mono- and asymmetric methyltransferase	Hmt1
KAF4306851.1	Protein serine/threonine kinase	Pkc1
KAF4304100.1	ATP-dependent RNA helicase of the SFI superfamily	Nam7
KAF4314396.1	Integral ER membrane protein with type-III transmembrane domains	Ice2
KAF4308353.1	Cyclin-dependent kinase	Pho85
KAF4314257.1	Subunit of the inner ring of the nuclear pore complex (NPC)	Nup188
KAF4303354.1	Subunit of the Ssh1 translocon complex	Ssh1
KAF4308602.1	Diacylglycerol kinase	Dgk1

Table S2. PCR primers used in this study.

Primer	Sequence (5'-3')[a]	Relevant characteristics
**Nem1-UP-F (P1)**<br>**Nem1-UP-R (P2)**	AAGTGTGCGGTGAAATCTGC<br>CAAAATAGGCATTGATGTGTTGACCTCCTGAAAG AGAAGCAGAATGAG	PCR primers to amplify *NEM1* upstream fragment for the construction of *NEM1* deletion mutants
**Nem1-DOWN-F (P3)**<br>**Nem1-DOWN-R (P4)**	CTCGTCCGAGGGCAAAGGAATAGAGTAGAGCATT TCTGTGGGGGTTCT<br>CTAACACAAACGAGACCAAC	PCR primers to amplify *NEM1* downstream fragment for the construction of *NEM1* deletion mutants
**Nem1-KO-F (P5)**<br>**Nem1-KO-R (P6)**	TTGGCGGCATCCTTTCCTTT<br>ACTCCTCACTTTCCGATAAC	PCR primers to amplify *NEM1* deletion fragment for the construction of *NEM1* deletion mutants
**Nem1-ID-F (P7)**<br>**Nem1-ID-R (P8)**	CGGTGGTTGTTGTTTTCGCT<br>CAAGCAGGAAGACGGCAATT	PCR primers for identification of *NEM1* deletion transformants
**Spo7-UP-F (P9)**<br>**Spo7-UP-R (P10)**	TCGGAAAAGAGAGTAAGAGT<br>CAAAATAGGCATTGATGTGTTGACCTCCATAGCC AGCACCGCAATACT	PCR primers to amplify *SPO7* upstream fragment for the construction of *SPO7* deletion mutants
**Spo7-DOWN-F (P11)**<br>**Spo7-DOWN-R (P12)**	CTCGTCCGAGGGCAAAGGAATAGAGTAGACACC CTTCTTAGTCTCCTC<br>GCTCTTCCCTCGCTATTGTG	PCR primers to amplify *SPO7* downstream fragment for the construction of *SPO7* deletion mutants
**Spo7-KO-F (P13)**<br>**Spo7-KO-R (P14)**	CTGGGTCGTATTCTCCTGAT<br>GAAGAAGACGAAATGGACAC	PCR primers to amplify *SPO7* deletion fragment for the construction of *SPO7* deletion mutants
**Spo7-ID-F (P15)**<br>**Spo7-ID-R (P16)**	ACTGGTTGTGCGTTATCGGC<br>TGATGAATGGTGGGAAGGAT	PCR primers for identification of *SPO7* deletion transformants

**Hph -F (P17)**<br>**Hph -R (P18)**	GGAGGTCAACACATCAATGCCTATT<br>CTACTCTATTCCTTTGCCCT	PCR primers for amplification of hygromycin resistance gene (*HPH*)
**Nem1-GFP-F (P19)**<br>**Nem1-GFP-R (P20)**	ACTCACTATAGGGCGAATTGGGTACTCAAATTGGTTCTCATCGCTGTCTTCATCCT<br>CACCACCCCGGTGAACAGCTCCTCGCCCTTGCTCACTGTTGCTTGCGGCTCACCGAG	PCR primers to amplify the native promoter region and open reading frame of *NEM1* for GFP fusion protein construction
**Nem1-GFP-ID-F (P21)**<br>**Nem1-GFP-ID-R (P22)**	CAACCTCATCATTTTCACGG<br>CGTGCTGCTTCATGTGGTCG	PCR Primer for PYF11-*NEM1*-GFP plasmid identification
**GFP-Spo7-F1 (P23)**<br>**GFP-Spo7-R1 (P24)**<br>**GFP-Spo7-F2 (P25)**<br>**GFP-Spo7-R2 (P26)**<br>**GFP-Spo7-F3 (P27)**<br>**GFP-Spo7-R3 (P28)**	ACTCACTATAGGGCGAATTGGGTACTCAAATTGGTTGTTCTCTCGCTTCTTGTTGT<br>TGAACAGCTCCTCGCCCTTGCTCACCATGGCTACTATCGCGGCTGCTT<br>ATGGTGAGCAAGGGCGAGGA<br>CTTGTACAGCTCGTCCATGC<br>GATCACTCTCGGCATGGACGAGCTGTACAAGATGTCCGAGTCGAAGCTCGAC<br>GAATGTTGAGTGGAATGATGCTACACCAAAGGAGTGGCGGAT	PCR primers to amplify the native promoter region and open reading frame of *SPO7* for GFP fusion protein construction
**GFP-Spo7-ID-F (P29)**<br>**GFP-Spo7-ID-R (P30)**	AGAAAACCCATCACCATCAC<br>CAAGAATGCGCATATGTGCG	PCR Primer for PYF11-GFP-*SPO7* plasmid identification
**Nem1-Flag-F (P31)**<br>**Nem1-Flag-R (P32)**	CTATAGGGCGAATTGGGTACTCAAATTGGTTCTCATCGCTGTCTTCATCCT<br>CTTTATAATCACCGTCATGGTCTTTGTAGTCTGTTGCTTGCGGCTCACCGAG	PCR primers to amplify the native promoter region and open reading frame of *NEM1* for 3×Flag fusion protein construction
**Nem1-Flag-ID-F (P33)**<br>**Nem1-Flag-ID-R (P34)**	CAACCTCATCATTTTCACGG<br>AGGAGCCTGAATGTTGAGTGG	PCR Primer for pHZ126-*NEM1*-3×Flag plasmid identification

**Nem1-Nub-F (P35)**	AATCAACTCACTAGTggatccATGAACTCGCTCAACATCCTC	PCR primers for amplification of the full cDNA sequence of the *NEM1* gene
**Nem1-Nub-R (P36)**	CCCAGATCTGAATTCggatccCTATGTTGCTTGCGGCTCAC	
**Nem1ΔCTR-Nub-F (P37)**	AATCAACTCACTAGTggatccATGAACTCGCTCAACATCCTC	PCR primers for amplification of the cDNA sequence of the *NEM1* gene lack of CTR
**Nem1ΔCTR-Nub-R (P38)**	CCCAGATCTGAATTCggatccGTTATCCGTGGGATCACTGAT	
**Spo7-Cub-F (P39)**	ACTGCAGGCGGCCGCactagtATGTCCGAGTCGAAGCTCGAC	PCR primers for amplification of the full cDNA sequence of the *SPO7* gene
**Spo7-Cub-R (P40)**	GATCCCCCCCGACATactagtCTACACCAAAGGAGTGGCGGA	
**Sec63-GFP-F (P41)**	ACTCACTATAGGGCGAATTGGGTACTCAAATTGGTTGGTGTGCTGACCACGGACACG	PCR primers to amplify the native promoter region and open reading frame of *SEC63*for GFP fusion protein construction
**Sec63-GFP-R (P42)**	CACCACCCCGGTGAACAGCTCCTCGCCCTTGCTCACCTCTTCATCGGTATCGGTATC	
**Sec63-GFP-ID-F (P43)**	TGAGCAAGGCAACCCGACCTA	PCR Primer for PYF11-*SEC63*-GFP plasmid identification
**Sec63-GFP-ID-R (P44)**	CGTGCTGCTTCATGTGGTCG	
**Ino1-UP-F (P45)**	AGCCACGTATTTGGATAATGA	PCR primers to amplify *INO1* upstream fragment for the construction of *INO1* deletion mutants
**Ino1-UP-R (P46)**	CCAAAATAGCATTGATGTGTTGACCTCCCTTGAAATCTCGAGTTGTCTA	
**Ino1-DOWN-F (P47)**	CTATCGCCTTCTTGACGAGTTCTTCTGAGCGTTTGTGCGCTCTGGCCAC	PCR primers to amplify *INO1* downstream fragment for the construction of *INO1* deletion mutants
**Ino1-DOWN-R (P48)**	CTCTGAGCGGGTAGGTGACGT	
**Ino1-KO-F (P49)**	ATCGCTCGGGCTTCAATTCGG	PCR primers to amplify *INO1* deletion fragment for the construction of *INO1* deletion mutants
**Ino1-KO-R (P50)**	TCGGTGCGTAGATGTTCACAA	

**Ino1-ID-F (P51)**<br>**Ino1-ID-R (P52)**	CCAGTTTGAAGGCCAGTCCTG<br>ATATGCTACAACATCATCACC	PCR primers for identification of *INO1* deletion transformants
**Neo-F (P53)**<br>**Neo-R (P54)**	GGAGGTCAACACATCAATGCT<br>TCAGAAGAACTCGTCAAGAAG	PCR primers for amplification of neomycin resistance gene (*NEO)*
**Pah1-UP-F (P55)**<br>**Pah1-UP-R (P56)**	TTGTGGTGGTTGATTGCGAT<br>CAAAATAGGCATTGATGTGTTGACCTCCCGGTAGGGCTCGTGGGTTTT	PCR primers to amplify *PAH1* upstream fragment for the construction of *PAH1*deletion mutants
**Pah1-DOWN-F (P57)**<br>**Pah1-DOWN-R (P58)**	CTCGTCCGAGGGCAAAGGAATAGAGTAGTAGGCGTTCTTCTTAGTTGC<br>TGTAGTCCTTGTGCTTCTCG	PCR primers to amplify *PAH1* downstream fragment for the construction of *PAH1* deletion mutants
**Pah1-KO-F (P59)**<br>**Pah1-KO-R (P60)**	GTAAGGAAGAAGAGGCGGCG<br>GAAAGGTTGTTGTAGAAAGC	PCR primers to amplify *PAH1* deletion fragment for the construction of *PAH1* deletion mutants
**Pah1-ID-F (P61)**<br>**Pah1-ID-R (P62)**	GCTCCTTTCCTCTCTCCACT<br>AGCAATACAGCTTGAGTCT	PCR primers for identification of *PAH1* deletion transformants
**INO1-RT-F (P63)**<br>**INO1-RT-R (P64)**	TGGTGATGACTTCAAGTCTGG<br>ACGGTGTTAGCCGCAACCATG	Quantitative real-time PCR primers for analysis of *INO1* expression level
**INO2-RT-F (P65)**<br>**INO2-RT-R (P66)**	ATGCTCGAGGCGAACTACAGG<br>CCTCGCCTTCTCAGCAGCCTG	Quantitative real-time PCR primers for analysis of *INO2* expression level
**OPI3-RT-F (P67)**<br>**OPI3-RT-R (P68)**	GGCACCTACCTGGGCGACTAC<br>GCAGCGCGATGCTGTACGCGA	Quantitative real-time PCR primers for analysis of *OPI3* expression level
**ACTIN-RT-F (P69)**<br>**ACTIN-RT-R (P70)**	TCCATCATGAAGTGCGATGTC<br>AATGGAACCACCGATCCAGAC	Quantitative real-time PCR primers for analysis of *BdACTIN* expression level
**Nem1-pYES2-F (P71)**<br>**Nem1-pYES2-R (P72)**	tcacactggcggccgctcgagATGAACTCGCTCAACATCCTC<br>ccctctagatgcatgctcgagCTATGTTGCTTGCGGCTCAC	PCR primers for amplification of the full cDNA sequence of the *NEM1*gene used for yeast complementation

**Pah1-Nub-F (P73)**<br>**Pah1-Nub-R (P74)**	AATCAACTCACTAGTggatccATGCAGTACGTCCGCGGGCTG<br>CCCAGATCTGAATTCggatccTTATCTCCGAGGAGTACTTGGC	PCR primers for amplification of the full cDNA sequence of the *PAH1* gene
**Pah1-Cub-F (P75)**<br>**Pah1-Cub-R (P76)**	ACTGCAGGCGGCCGCactagtATGCAGTACGTCCGCGGGCTG<br>GATCCCCCCCGACATactagtTTATCTCCGAGGAGTACTTGGC	PCR primers for amplification of the full cDNA sequence of the *PAH1* gene
**Pah1-GFP-F (P77)**<br>**Pah1-GFP-R (P78)**	ACTCACTATAGGGCGAATTGGGTACTCAAATTGGTTTACTGGCGGCTGTTTTGCGG<br>CACCACCCCGGTGAACAGCTCCTCGCCCTTGCTCACTCTCCGAGGAGTACTTGGCTC	PCR primers to amplify the native promoter region and open reading frame of *PAH1*for GFP fusion protein construction
**Pah1-GFP-ID-F (P79)**<br>**Pah1-GFP-ID-R (P80)**	CAGCGAAAGCAGCGAAAGTT<br>CGTGCTGCTTCATGTGGTCG	PCR Primer for PYF11-*PAH1*-GFP plasmid identification
**Pah1-Flag-F (P81)**<br>**Pah1-Flag-R (P82)**	CTATAGGGCGAATTGGGTACTCAAATTGGTTTACTGGCGGCTGTTTTGCGG<br>CTTTATAATCACCGTCATGGTCTTTGTAGTCTCTCCGAGGAGTACTTGGCTC	PCR primers to amplify the native promoter region and open reading frame of *PAH1* for 3×Flag fusion protein construction
**Pah1-Flag-ID-F (P83)**<br>**Pah1-Flag-ID-R (P84)**	CAGCGAAAGCAGCGAAAGTT<br>AGGAGCCTGAATGTTGAGTGG	PCR Primer for pHZ126-*PAH1*-3×Flag plasmid identification
**ChIp-INO1-ORF-F (P85)**<br>**ChIp-INO1-ORF-R (P86)**	AGGAATCATCCGTGGTTGAG<br>CTGCACGCGGTGCATGTGCA	ChIp-qPCR primers for analysis of Pah1-GFP enrichment in ORF of *INO1*
**ChIp-INO1-Pro-F (P87)**<br>**ChIp-INO1-Pro-R (P88)**	CAGCGAAAGCAGCGAAAGTT<br>CGTGCTGCTTCATGTGGTCG	ChIp-qPCR primers for analysis of Pah1-GFP enrichment in promoter of *INO1*

**ChIp-INO2-ORF-F (P89)**<br>**ChIp-INO2-ORF-R (P90)**	GATGTACGTAATTGCCTGCC<br>GCTTCTGGGCGGATGTGCCC	ChIp-qPCR primers for analysis of Pah1-GFP enrichment in ORF of *INO2*
**ChIp-INO2-Pro-F (P91)**<br>**ChIp-INO2-Pro-R (P92)**	TTGACGGAGTTTGTCACCGC<br>CCGAAGAGGAGCTGAAGGAG	ChIp-qPCR primers for analysis of Pah1-GFP enrichment in promoter of *INO2*
**ChIp-OPI3-ORF-F (P93)**<br>**ChIp-OPI3-ORF-R (P94)**	GTACTGCAATTCTCTCTTCT<br>TTGCGGGTTTGTGGGCTCC	ChIp-qPCR primers for analysis of Pah1-GFP enrichment in ORF of *OPI3*
**ChIp-OPI3-Pro-F (P95)**<br>**ChIp-OPI3-Pro-R (P96)**	GCTTGTAGGCGATTCAAAGC<br>ACTGTCCGCGAAGACAATAG	ChIp-qPCR primers for analysis of Pah1-GFP enrichment in promoter of *OPI3*
**ChIp-GAPDH-Pro-F (P97)**<br>**ChIp-GAPDH-Pro-F (P98)**	GAAAATGCTCATCTTGGGTC<br>TCTCTGAAGACCGAAGCGAA	ChIp-qPCR primers for analysis of Pah1-GFP enrichment in promoter of *GAPDH*

[a] The respective restriction enzyme sites included in primers are listed in lowercase in the sequence.
